# Novel roles of luteinizing hormone (LH) in tissue regeneration-associated functions in endometrial stem cells

**DOI:** 10.1038/s41419-022-05054-7

**Published:** 2022-07-13

**Authors:** Se-Ra Park, Seong-Kwan Kim, Soo-Rim Kim, Jeong-Ran Park, Soyi Lim, In-Sun Hong

**Affiliations:** 1 Department of Health Sciences and Technology, GAIHST, Gachon University, Incheon, 21999 Republic of Korea; 2 Department of Molecular Medicine, School of Medicine, Gachon University, Incheon, 406-840 Republic of Korea; 3 Division of Science Education, Kangwon National University, Chuncheon, 24341 Republic of Korea; 4 Department of Obstetrics and Gynecology, Gachon University Gil Medical Center, Incheon, Republic of Korea

**Keywords:** Reproductive disorders, Adult stem cells

## Abstract

Luteinizing hormone (LH) stimulates the synthesis and secretion of the key steroid hormone estrogen, which subsequently promotes ovarian follicular growth and development. Therefore, the administration of exogenous LH to achieve superovulation (multiple ovulations) and an LH surge is commonly used as the most effective therapeutic option in a majority of in vitro fertilization (IVF) clinics. However, a relatively low pregnancy rate (between 20% and 35%) is one of the most challenging aspects of LH-based infertility treatment. Furthermore, the major cause of this low pregnancy rate in LH-based infertility treatment remains unidentified. Recent studies have shown that endometrial stem cell loss or deficiency can significantly decrease tissue regeneration ability during the menstrual cycle and reduce endometrial receptivity. In this context, we postulated that the low pregnancy rates following LH-based ovarian hyperactivation may be the result of the adverse effects of consecutive exogenous LH administration on endometrial stem cells. To the best of our knowledge, this study revealed for the first time that in addition to its previously reported roles in stimulating ovarian functions through the pituitary-gonadal axis, LH brings about the extragonadal suppression of various tissue regeneration-associated functions in endometrial stem cells, such as self-renewal, migration ability, multilineage differentiation potential, and pluripotency/stemness, by inhibiting pro-survival Akt and ERK1/2 signaling pathways in vitro and in vivo, and as a consequence, it decreases the endometrial receptivity.

## Introduction

During folliculogenesis, luteinizing hormone (LH) promotes the production of a key steroid hormone, estrogen, in ovarian follicles, which subsequently stimulates ovarian follicular growth and maturation [1, 2]. Therefore, long exogenous LH agonist administration protocols are frequently used as the key drivers of ovarian follicle growth and maturation in most assisted reproductive technologies (ARTs) [2, 3]. However, the rate of successful implantation and subsequent pregnancy outcomes in patients with infertility following consecutive LH administration is low and ranges between 20% and 35% [4–6]. Therefore, it is necessary to identify the reason for the low success rate of pregnancies following the employment of LH-based ovarian hyperactivation procedures in infertility treatment.

The endometrium, the mucosal inner lining of the uterine cavity, is one of the most highly regenerative tissues, which undergoes >400 cycles of growth and shedding during the female reproductive life [7, 8]. Endometrial receptivity (thickness between 8 and 11 mm) is a major limiting factor responsible for embryo implantation and subsequent successful pregnancy outcomes [9]. Poor endometrial receptivity (thickness less than 7 mm) is a major cause of the recurrent implantation failures or repeated miscarriages observed among patients undergoing infertility treatment [10, 11]. Similar to other rapidly remodeling tissues, resident stem cells play a crucial role in the cyclic regeneration and repair of damaged endometrium during each menstrual cycle [12, 13]. Consistent activation and recruitment of endometrial stem cells that can differentiate into multiple endometrial cell types are necessary to increase endometrial receptivity and the subsequent chances of pregnancy [14]. Lucas et al. recently observed that endometrial stem cell loss or deficiency can significantly reduce endometrial receptivity and subsequently lead to preterm delivery or miscarriage [14]. Similarly, Tomari et al. found that the expressions of somatic stem cell-related genes ABCG2 and ALDH1A1 were significantly decreased in endometrial cells isolated from non-receptive patients compared with receptive women [15]. They also showed markedly increased cellular senescence and cell cycle arrest in endometrial stromal cells originating from non-receptive patients [15]. In addition, enhanced cell proliferation of endometrial cells was positively correlated with endometrial receptivity [16].

Importantly, the functional LH receptor (LHR), which was previously considered to be gonad tissue- or cell-specific, is highly expressed in the extragonadal endometrial tissue throughout the menstrual cycle [17, 18]. It was recently observed that LH affects the uterine endometrium by promoting the differentiation of endometrial fibroblasts into fully differentiated decidual cells, which are essential for embryo implantation [19]. Therefore, studies on extragonadal LH exposure in the endometrium provide new insights into the possible adverse effects of LH administration during infertility treatment on endometrial receptivity, which is primarily supported by the local endometrial stem cells. Although, LH is well-known to stimulate ovarian follicular development by inducing steroid hormone production and thus subsequently indirectly increasing female fertility [20, 21], its direct effect on endometrial receptivity still remains controversial. Recently, several studies have revealed that LH has no significant effect on pregnancy rate or is associated with decreased endometrial receptivity. Indeed, Khoury et al. found that LH rise during artificial embryo transfer cycles does not alter actual pregnancy outcomes [22]. Similarly, Jarvela et al. also observed that endometrial volume significantly reduced after the human choriogonadotropin (hCG) analog, known as LH, treatment [23]. Thus, we hypothesized that in addition to its previously reported roles in stimulating ovarian functions through the pituitary-gonadal axis, consecutive administration of LH for ovarian hyperactivation could directly inhibit tissue regeneration-associated functions in endometrial stem cells, which in turn would reduce endometrial receptivity. Importantly, this could be the reason for the low success rate in pregnancies achieved through LH-based infertility treatment. Moreover, the direct extragonadal effects of LH on the tissue regeneration-associated functions in endometrial stem cells and the exact molecular mechanisms underlying these events remain unknown.

To the best of our knowledge, it is demonstrated for the first time in the present study that in addition to its previously reported roles, LH extragonadally inhibits tissue regeneration-associated functions in endometrial stem cells, such as self-renewal, migration capacity, multilineage differentiation potential, and pluripotency/stemness, both in vitro and in vivo. In addition, LH inhibits the crucial pro-survival signaling cascades Akt and ERK1/2, and the inhibition of these signaling components using specific inhibitors significantly abolishes the adverse effects on endometrial stem cell functions induced by LH. These findings may facilitate our understanding of the mechanism of action of extragonadal LH and aid in the development of better therapeutic options by alleviating infertility treatment-induced adverse effects on endometrial receptivity.

## Results

### LH suppresses tissue regeneration-associated functions in human endometrial stem cells

**Fig. 1:**
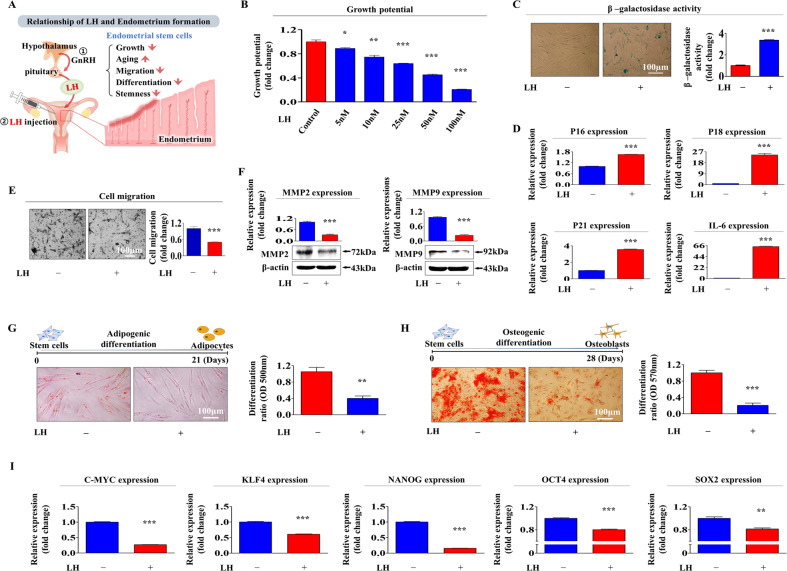
LH treatment decreases self-renewability, migration, transdifferentiation abilities, pluripotency/stemness of human endometrial stem cells. We postulated that LH would inhibit tissue regeneration-associated functions in the endometrial stem cells (**A**). The inhibitory effects of LH on the self-renewability of endometrial stem cells were determined at 72 h after LH (5, 10, 25, 50, and 100 nM) treatment using MTT-based assays. Cell growth rates were estimated as the relative viability (%) of LH-treated groups compared to that in vehicle-treated groups (**B**). Effects of LH treatment on the cellular aging of endometrial stem cells were determined by evaluating the relative activities of senescence-associated β-galactosidase (SA-β-Gal) after continuous subculture with or without LH (25 nM) treatment (**C**). Effects of LH treatment on the mRNA expression levels of several cellular aging-associated genes (*p16* ^*INK4a*^, *p18* ^*INK4c*^, *p21* ^*Cip1*^, and *IL-6)* were also evaluated by performing qPCR (**D**). Suppressive effects of LH on the migration potential of endometrial stem cells were assessed after LH treatment (25 nM) for 72 h using transwell assays. A clear reduction in the migration ability across the transwell membranes (8.0 μm pores) was observed following LH treatment (**E**). Effects of LH treatment on MMP-2 and MMP-9 expression levels measured using western blotting (**F**). The suppressive effects of LH (25 nM) on in vitro differentiation abilities of endometrial stem cells into adipocytes (**G**) and osteoblasts (**H**) were evaluated by performing oil red O staining and alizarin red S staining, respectively, after 2 weeks of differentiation. Suppressive effects of LH (25 nM) on the expression of various multipotent capacity-related factors (*C-MYC*, *KLF4*, *NANOG*, *OCT4*, and *SOX2*) were evaluated by performing qPCR (**I**). β-actin was used as an internal control to normalize protein expression. PPIA was used as an internal control to normalize mRNA expression for qPCR analysis. All experiments were performed in triplicates. Data are presented as mean ± standard deviation (SD). *p < 0.05; **p < 0.005; and ***p < 0.001 (two-sample *t* test).

Human endometrial stem cells were isolated from hysterectomy samples and properly cultured as described in our previous studies [24–28] (Suppl. Fig. 1A). The pluripotency of isolated cells was assessed by flow cytometry using various negative (CD44 and CD45) and positive (CD34, CD73, CD105, CD140b, CD146, and susD2) surface markers (Suppl. Fig. 1B). Additionally, their multilineage differentiation capacities into other cell types were analyzed by inducing adipocytes (Suppl. Fig. 1C) and osteoblasts (Suppl. Fig. 1D) differentiation. A schematic diagram describing the suppressive effects of LH on various endometrial stem cell functions is shown in Fig. 1A. By inhibiting various beneficial functions of endometrial stem cells, we investigated whether LH suppresses endometrial receptivity and observed that LH remarkably inhibited the self-renewal ability of endometrial stem cells in a dose-dependent manner (Fig. 1B). The enhanced activity of senescence-associated beta-galactosidase (SA-β-Gal) is a commonly used in vitro biomarker for cellular aging [29]. Therefore, endometrial stem cells were consecutively passaged with or without LH treatment to assess whether LH treatment could accelerate cellular aging (senescence) (Fig. 1C). In addition to the increases in the SA-β-Gal levels, enhanced levels of cell cycle regulatory proteins or cytokines, such as p16^INK4A^, p18, p21^CIP1^, and IL-6, have been widely used as reliable cellular aging (senescence) biomarkers [30]. The expression of these cellular aging-related proteins was markedly elevated following LH treatment (Fig. 1D). LH also significantly reduced the migratory capacity of the endometrial stem cells (Fig. 1E). The expression levels of MMP-2 and -9 with or without LH treatment were also analyzed to further assess their inhibitory effect on the migratory capacity, as they are known regulators of cell migration (Fig. 1F). Moreover, LH treatment lowered the multilineage differentiation ability to convert endometrial stem cells into adipocytes (Fig. 1G) and osteoblasts (Fig. 1H). LH also clearly reduced the mRNA expression levels of pluripotency/stemness-related factors, such as *C-MYC*, *KLF4*, *NANOG*, *OCT4*, and *SOX2* (Fig. 1I).

### LH treatment reduces metabolic functions in endometrial stem cells

**Fig. 2:**
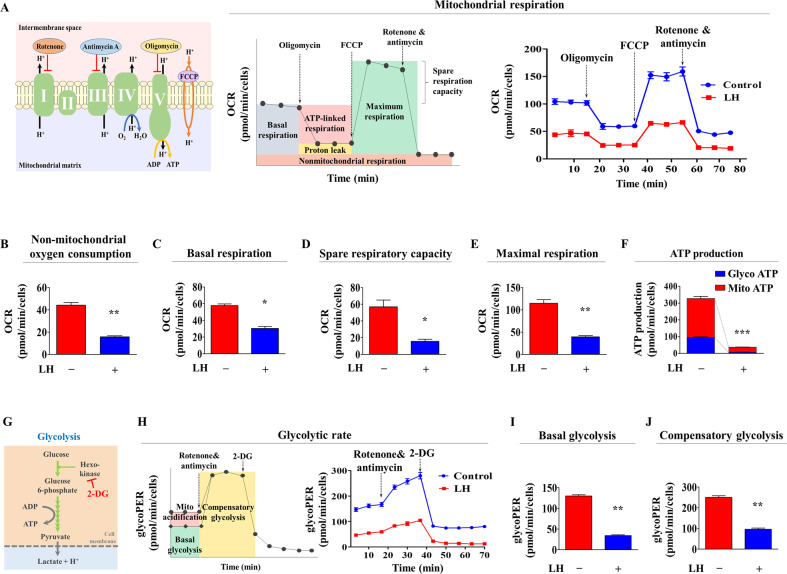
LH treatment triggers energy metabolic switching in endometrial stem cells. To evaluate the effect of LH treatment on energy metabolism in endometrial stem cells, oxidative phosphorylation or glycolysis were determined after treating or not treating cells with LH (25 nM) for 72 h. Oxidative phosphorylation profiles in the mitochondria were evaluated by monitoring oxygen (O_2_) consumption rates (OCR) simultaneously using a Seahorse XF flux analyzer (Seahorse Biosciences) (**A**). Cells were seeded at a density of 20,000 cells per well density in multi-well plates containing the growth medium. The seeded cells were then treated with 1.5 μM oligomycin ATP synthase inhibitor (also known as a complex V blocker) to inhibit coupled respiration (respiration related to ATP synthesis), 2 μM FCCP to disrupt the proton gradient (Δψm) across the mitochondrial membrane, 0.5 uM rotenone, and 0.5 uM antimycin A to completely collapse mitochondrial electron transport. These inhibitors were automatically injected into each well, and real-time OCR was recorded every 15 mins. LH treatment decreased the overall mitochondrial respiration capacity and nonmitochondrial OCR of endometrial stem cells (**B**). LH treatment also suppressed the levels of basal mitochondrial respiration potential (**C**), spare respiratory capacity (**D**), and maximal respiratory capacity (**E**). ATP production decreased remarkably upon LH treatment in both the cytosol and mitochondria (**F**). Glycolytic rates of endometrial stem cells are determined by measuring the real-time OCR and ECAR (extracellular acidification rate) to analyze glycolytic proton efflux rates (glycoPER) following various inhibitors treatments. Briefly, cells were incubated in a glucose-free medium with 1.67 μM antimycin A, rotenone, and 50 mM 2-deoxyglucose (2-DG, glycolytic inhibitor) treatments (**G**). Compensatory glycolysis is the glycolytic capacity of cells after preventing mitochondrial respiration and compensating ATP production through glycolysis to meet sudden energy demand. The overall glycolytic capacity of the endometrial stem cells significantly decreased upon LH treatment (25 nM) for 72 h (**H**). LH treatment also decreased basal (**I**) and compensatory (**J**) glycolysis. Glycolytic ECAR measurements were normalized by the cell numbers in each well. Bar graphs represent the averages of three independent experiments. Significant differences are presented as *p < 0.05, **p < 0.005, and ***p < 0.001 (two-sample *t* test).

Metabolic activities, such as proliferation, differentiation, and pluripotency/stemness, are regulated through mitochondrial oxidative phosphorylation or cytosolic glycolysis [31–33]. Moreover, various hormones can cause changes in the energy-producing (metabolic) activities in multiple cell types [34, 35]. Oxidative phosphorylation in oxygen-consuming mitochondria is a key index of sustainable energy (ATP)-producing capacity and overall cellular health [36]. Thus, to analyze the potential effect of LH on the metabolic activities of endometrial stem cells, overall oxidative phosphorylation levels with or without LH treatment were assessed using the Seahorse XF analyzer, which provides accurate quantitative information on mitochondrial respiration by monitoring real-time oxygen consumption rates (OCR) in living cells [37]. The specific ATP synthase blocker oligomycin (also known as an inhibitor of complex V of the electron transport chain) was injected to prevent coupled mitochondrial respiration [38]. FCCP (a potent oxidative phosphorylation uncoupler) was injected to disrupt mitochondrial membrane potential (Δψm), inducing proton leak across the inner membrane of the mitochondria, thus allowing oxygen consumption with no ATP production [36]. Therefore, FCCP treatment can be used to measure the real-time maximal respiration (oxygen consumption) potential of the mitochondria. The mitochondrial oxidative phosphorylation of endometrial stem cells was significantly decreased by LH treatment (Fig. 2A), with reduced non-mitochondrial OCR (Fig. 2B). LH treatment also clearly decreased basal respiration (Fig. 2C), spare respiration (Fig. 2D), and maximal respiratory capacity (Fig. 2E), which are used to measure the extra amount of ATP production that can be synthesized by mitochondrial respiration to adjust a sudden increase in ATP (energy) demand [39]. In addition, overall ATP production from both the mitochondria and cytosol significantly decreased following LH treatment (Fig. 2F). Because glycolysis produces pyruvic acid and protons from glucose under aerobic conditions [40, 41], we quantitatively measured the glycolytic rates by analyzing the real-time extracellular acidification rate (ECAR) (Fig. 2G). A schematic diagram describing glycolytic activity using a Seahorse XF analyzer is shown in Fig. 2H. The glucose analog 2-deoxyglucose was injected to prevent glycolysis and thus provides a real-time measurement of basal ECAR [42]. The mitochondrial respiratory chain complexes I and III were completely inhibited by rotenone and antimycin A, respectively, thereby blocking mitochondrial oxidative phosphorylation [43]. The real-time information on the glycolytic rates showed that LH-treated endometrial stem cells showed significantly lower glycolytic activity than untreated cells (Fig. 2H). LH treatment also markedly decreased basal glycolysis (Fig. 2I) and compensatory glycolytic rates (Fig. 2J).

### LH exerts inhibition on tissue regeneration-associated functions through LHR

**Fig. 3:**
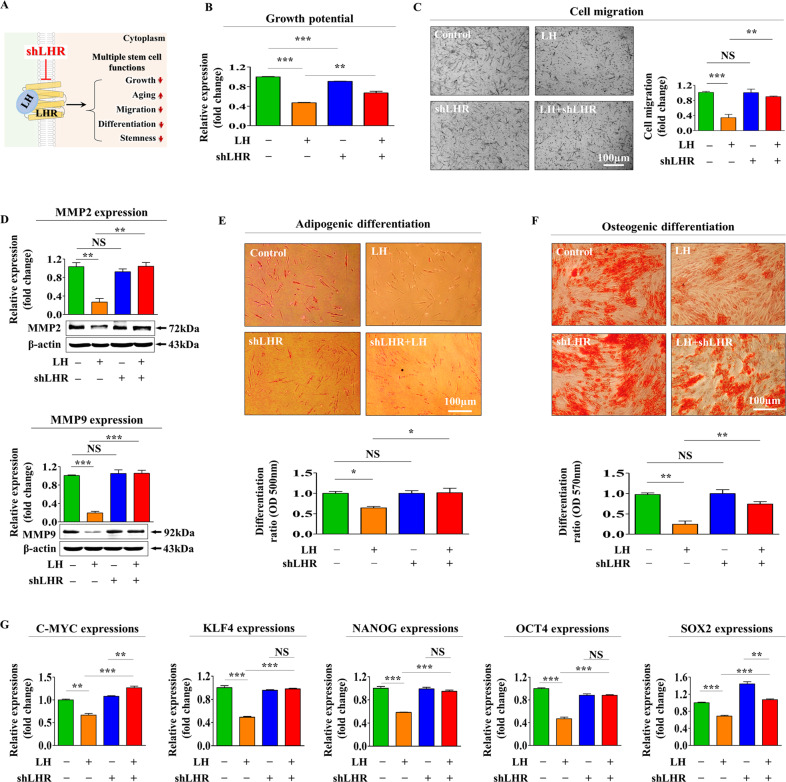
LH receptor (LEHR) mediates adverse effects of LH on tissue regeneration-associated functions in endometrial stem cells. A schematic diagram showing the role of LHR in controlling LH-mediated adverse effects on various endometrial stem cell functions are described (**A**). Endometrial stem cells were treated with 25 nM LH alone or simultaneously transfected with an LHR targeting shRNA. Their effects on the self-renewability of endometrial stem cells were assessed using MTT assays (**B**). LHR depletion attenuates LH-induced changes in cell migration potential determined using transwell assays (**C**) and western blotting for MMP-2 and MMP-9 (**D**). LHR knockdown alleviates the inhibitory effects of LH on endometrial stem cells into the differentiation of adipocytes (**E**) and osteoblasts (**F**) as determined by oil red O staining and alizarin red S staining, respectively. LHR depletion attenuates the mRNA expression levels of multipotency-associated genes *C-MYC*, *KLF4*, *NANOG*, *OCT4*, and *SOX2*, as measured using qPCR (**G**). β-actin was used as an internal control to normalize protein expression. PPIA was used as an internal control to normalize mRNA expression for qPCR analysis. All experiments were performed in triplicates. Data are presented as mean ± standard deviation (SD). *, *p* < 0.05; **, *p* < 0.005; and ***, *p* < 0.001 (two-sample *t* test).

LH is known to exert its effect through its cognate receptor LHR, which belongs to the G-protein coupled receptor superfamily [35, 44]. To investigate whether LH exerts its effects through the cognate receptor LHR, its expression was successfully knocked out by transfecting endometrial stem cells with a specific LHR shRNA (Suppl. Fig. 2A–C). A schematic diagram summarizing the functions of LHR that mediates LH-induced suppressive effects on various endometrial stem cell functions is shown in Fig. 3A. The LH-mediated inhibitory effect on self-renewal ability was abolished by LHR depletion (Fig. 3B). Moreover, LHR knockdown significantly attenuated the LH-induced inhibitory effects on migratory potential (Fig. 3C) and the expression of *MMP-2* and *MMP-9* (Fig. 3D). LH-induced suppressive effects on differentiation potential into adipocytes (Fig. 3E) and osteoblasts (Fig. 3F) were also significantly abolished by LHR depletion. Similarly, LH-induced inhibitory effects on the expression of various pluripotency-associated genes, such as C-*MYC*, *KLF4*, *NANOG*, *OCT4*, and *SOX2*, were remarkably reduced by LHR knockdown (Fig. 3G).

### LH-induced inhibitory effects are mediated through Akt and ERK1/2 signaling

**Fig. 4:**
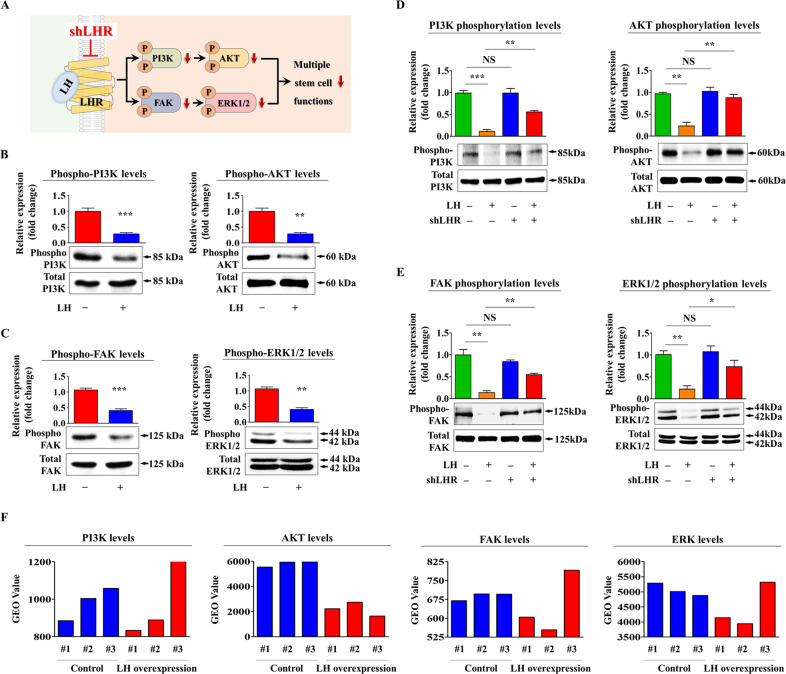
LH-induced adverse effects on various endometrial stem cell functions are mediated through ERK1/2 or Akt signaling pathway. A schematic diagram describing the roles of PI3K/Akt or FAK/ERK1/2 signaling in controlling LH-induced adverse effects is shown (**A**). Endometrial stem cells were treated with or without 25 nM LH for 10 min. LH-induced changes in Akt, ERK1/2, FAK, and PI3K signaling activities were examined using western blotting (**B**, **C**). Next, cells were treated with 25 nM LH alone or simultaneously transfected with an LHR targeting shRNA. Subsequent changes in these signaling activities were examined using western blotting (**D**, **E**). In addition, information in the GEO data repository was used to analyze the interconnections between LH overexpression and the relative expressions of PI3K, AKT2, FAK, or ERK (**F**). β-actin was used as an internal control to normalize protein expression. All experiments were performed in triplicates. Data are presented as mean ± standard deviation (SD). *, *p* < 0.05; **, *p* < 0.005; and ***, *p* < 0.001 (two-sample *t* test).

**Fig. 5:**
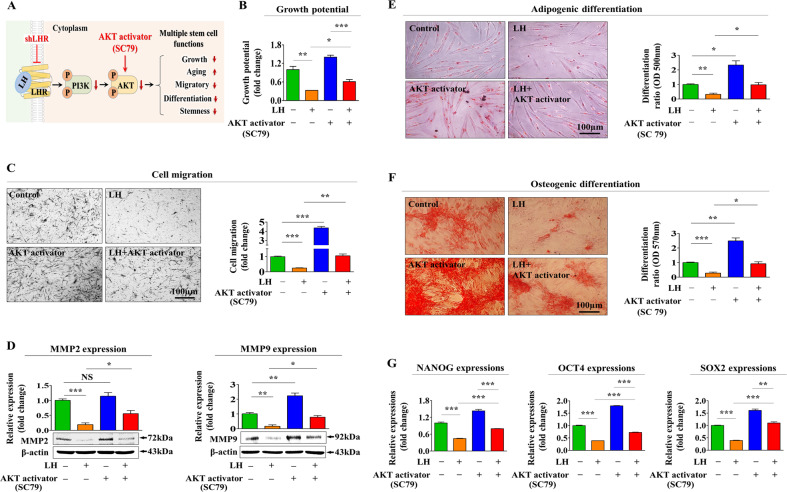
Activation of Akt signaling pathway using a synthetic activator alleviates LH-induced adverse effects on endometrial stem cells. A schematic diagram describing the role of the PI3K/Akt signaling in controlling LH-mediated adverse effects on various endometrial stem cell functions is shown (**A**). Cells were prestimulated with 10 µM Akt phosphorylation activator SC79 for 1 h before 25 nM LH treatments for 48 h. LH-induced adverse effects on the self-renewability of endometrial stem cells were then evaluated using MTT-based assays (**B**). Akt activator SC79 treatment alleviates the LH-mediated inhibitory effects on migration potential as determined using transwell assays (**C**) and western blotting for MMP-2 and MMP-9 (**D**). Cells were prestimulated with 10 µM Akt activator SC79 for 1 h before 25 nM LH treatment for 48 h. Akt activator SC79 treatment alleviates the LH-mediated inhibitory effects of adipocyte (**E**) and osteoblast **G** differentiation abilities as demonstrated using oil red O and alizarin red S staining, respectively. Akt phosphorylation activator SC79 (10 µM) alleviates the LH-mediated changes in the mRNA expression levels of multipotency-associated genes *C-MYC*, *KLF4*, *NANOG*, *OCT4*, and *SOX2* as evaluated using qPCR (**G**). β-actin was used as an internal control to normalize protein expression. PPIA was used as an internal control to normalize mRNA expression for qPCR analysis. All experiments were performed in triplicates. Data are presented as mean ± standard deviation (SD). *, *p* < 0.05; **, *p* < 0.005; and ***, *p* < 0.001 (two-sample *t* test).

**Fig. 6:**
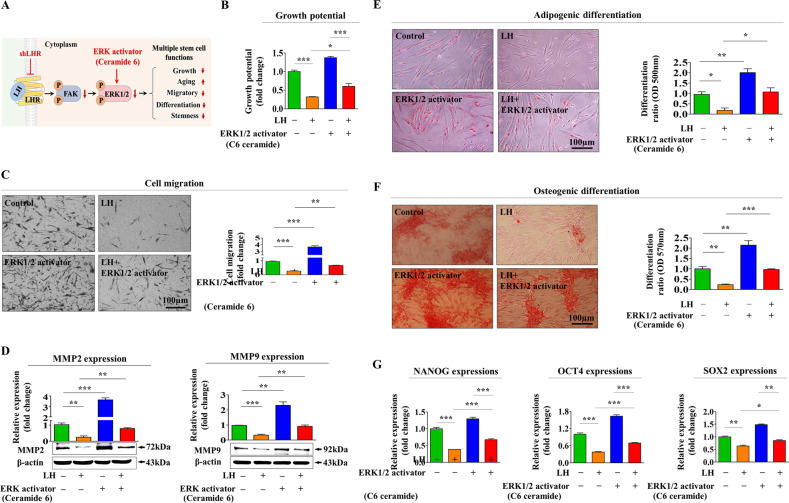
Activation of ERK1/2 signaling pathway using a synthetic activator alleviates LH-induced adverse effects on endometrial stem cells. A schematic diagram describing the role of the FAK/ERK1/2 signaling in controlling LH-mediated adverse effects on various endometrial stem cell functions is shown (**A**). Cells were prestimulated with 10 µM ERK1/2 phosphorylation activator ceramide C6 for 1 h before 25 nM LH treatments for 48 h. LH-induced adverse effects on the self-renewability of endometrial stem cells were evaluated using MTT-based assays (**B**). ERK1/2 activator ceramide C6 treatment alleviates LH-mediated inhibitory effects on migration potential as demonstrated using transwell assays (**C**) and western blotting for MMP-2 and MMP-9 (**D**). Cells were prestimulated with 10 µM ERK1/2 activator ceramide C6 for 1 h before 25 nM LH treatment for 48 h. ERK1/2 activator ceramide C6 treatment alleviates the LH-mediated inhibitory effects on adipocyte (**E**) and osteoblast **F** differentiation abilities as examined by oil red O and alizarin red S staining, respectively. ERK1/2 phosphorylation activator ceramide C6 (10 µM) alleviated the LH-mediated changes in the mRNA expression levels of multipotency-associated genes *C-MYC*, *KLF4*, *NANOG*, *OCT4*, and *SOX2* as demonstrated using qPCR (**G**). β-actin was used as an internal control to normalize protein expression. PPIA was used as an internal control to normalize mRNA expression for qPCR analysis. All experiments were performed in triplicates. Data are presented as mean ± standard deviation (SD). *, *p* < 0.05; **, *p* < 0.005; and ***, *p* < 0.001 (two-sample *t* test).

To investigate the molecular mechanisms underlying the inhibitory effects of LH on tissue regeneration-associated functions, we assessed the effects of LH on PI3K/Akt and FAK/ERK1/2 signaling cascades known to be involved in self-renewability [45], multilineage differentiation capacity [46], and migration potential [47] of various stem cells. A schematic summary describing the roles of PI3K/Akt and FAK/ERK1/2 signaling pathways in LH-mediated inhibitory effects is shown in Fig. 4A. We examined whether PI3K/Akt (Fig. 4B) and FAK/ERK1/2 (Fig. 4C) signaling activities were suppressed following LH exposure. We further assessed the effect of LHR knockdown on LH-induced inhibition of these signaling pathways. Interestingly, LHR knockdown significantly attenuated the LH-mediated suppressive effects on PI3K/Akt (Fig. 4D) and FAK/ERK1/2 (Fig. 4E) signaling activities. Moreover, information in the Gene Expression Omnibus (GEO), a public functional genomics data repository, indicated that these signaling activities generally decreased upon LH overexpression (Fig. 4F). To further investigate whether these signaling activities could mediate LH-induced inhibitory effects on tissue regeneration-associated functions, we examined the effects induced by LH on endometrial stem cells with or without Akt activator SC79 (Fig. 5A) or ERK1/2 activator ceramide C6 (Fig. 6A) pretreatment. Importantly, the LH-mediated suppressive effects on self-renewal were clearly attenuated upon SC79 (Fig. 5B) or ceramide C6 (Fig. 6B) pretreatment. Consistent with these findings, LH-induced suppressive effects on the migration potential and *MMP*-2/9 expression were also significantly attenuated by SC79 (Fig. 5C, D) or ceramide C6 (Fig. 6C, D) pretreatment. SC79 (Fig. 5E–G) or ceramide C6 (Fig. 6E–G) prestimulation clearly abolished the suppressive effects mediated by LH on multilineage differentiation abilities into adipocytes and osteoblasts as well as the mRNA expression levels of various pluripotency/stemness-associated factors, such as *NANOG*, *OCT4*, and *SOX2*.

### Proteome analysis of LH-mediated suppression of growth factors in endometrial stem cells

**Fig. 7:**
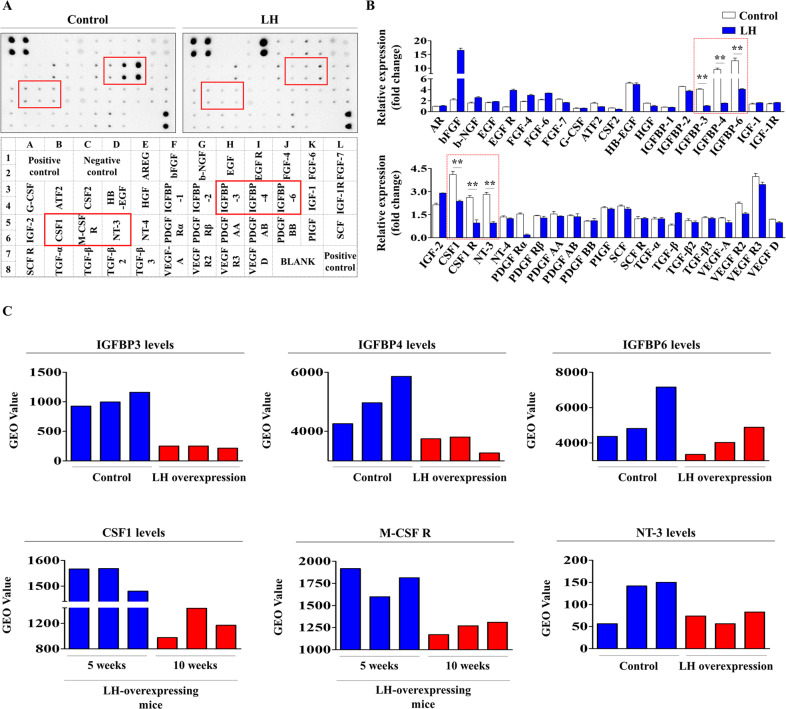
LH treatment reduces the secretion of multiple protein factors related to tissue regeneration-associated signaling pathways. A human growth factor antibody array (membrane-based sandwich immunoassay) was conducted using culture medium samples from untreated or 25 nM LH-treated endometrial stem cells. Forty different antibodies specific for various growth factors, chemokines, and receptors were spotted in duplicate on nitrocellulose membranes. Six protein factors (insulin-like growth factor-binding protein [IGFBP] 3, IGFBP4, IGFBP6, CSF1, CSF1R, and NT3) were remarkably reduced in culture medium samples from LH-treated cells (**A**, **B**). In addition, information on the GEO public data repository was analyzed to examine the correlations between reduced expressions of these six protein factors and LH expression itself (**C**). All experiments were performed in triplicates. Data are presented as mean ± standard deviation (SD). *, *p* < 0.05; **, *p* < 0.005; and ***, *p* < 0.001 (two-sample *t* test).

To investigate the major secreted protein components related to the inhibitory effects of LH on various endometrial stem cell functions, we examined the effects of LH on the secretion of various growth factors or cytokines using membrane-based multiple antibody arrays. We detected secretory changes in 40 different proteins in the endometrial stem cells in response to LH treatment. The secretion of six prominent factors [insulin-like growth factor binding protein (IGFBP) 3, 4, 6, colony-stimulating factor 1 (CSF1), colony-stimulating factor 1 receptor (CSF1R), and neurotrophin 3 (NT3)] associated with Akt and ERK1/2 signaling activities was significantly decreased upon LH exposure, whereas only minor changes were detected for other protein secretions (Fig. 7A, B). These results indicate that reduced secretion of these proteins might be at least partially associated with the LH-induced suppressive effect on Akt and ERK1/2 signaling activities and the subsequent inhibition of tissue regeneration-associated functions. In addition, information in the GEO data repository showed that the expression levels of these six prominent proteins decreased upon LH overexpression (Fig. 7C). To further investigate whether these signaling activities could regulate the expressions of these six prominent factors, we examined expression levels of these factors using real-time PCR with or without Akt inhibitor (inhibitor V) or ERK1/2 inhibitor PD98059 pretreatment (Suppl. Fig. 3). Interestingly, the levels of these proteins were significantly decreased by inhibitor V or PD98059 treatment only. In addition, some synergistic effects were also observed when LH and these inhibitors were treated simultaneously (Suppl. Fig. 3). These results suggest that the reduced secretion of six prominent factors might serve as potential downstream targets of Akt and ERK1/2 signaling activities and mediate the suppressive effect of LH on endometrial stem cells.

### LH inhibits tissue regeneration-associated functions of endometrial stem cells and subsequently suppresses tissue repair of damaged endometrium in vivo

**Fig. 8:**
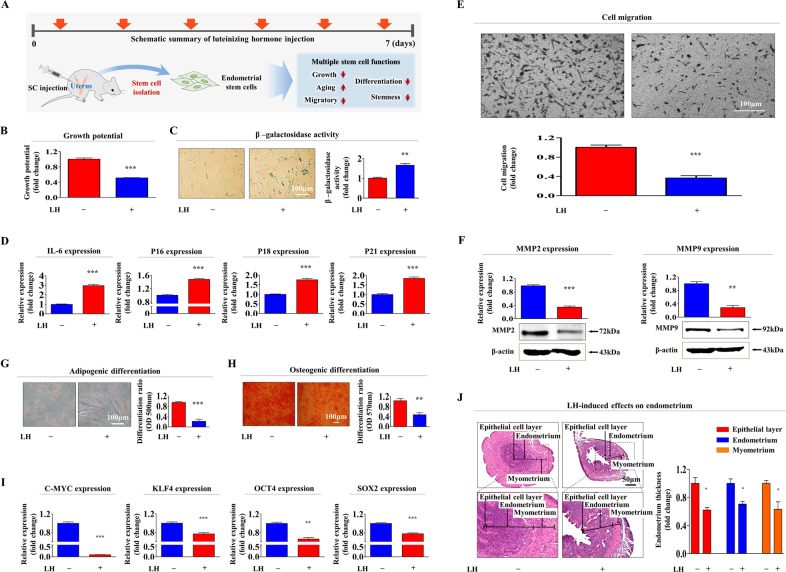
Consecutive LH administration remarkably inhibits tissue regeneration-associated functions in endometrial stem cells in vivo. A schematic summary of the overall in vivo experimental procedure as shown in ‘Materials and Methods’ is described **(A)**. Each mouse was administered with LH (1 µM/mouse, daily for 7 consecutive days) via intravenous injection through the tail vein. Next, endometrial stem cells were successfully obtained from the uterine tissues of mice using our previously established primary culture protocol. During in vitro expansion, isolated cells were then cultured either under continuous LH (25 nM) exposure or non-exposure conditions for mimicking the in vivo environment of consecutive LH administration. Their effects on the self-renewability of endometrial stem cells were determined using MTT-based assays. Cell growth rates were estimated as the relative viability of LH-treated groups as a percentage (%) of vehicle-treated groups (**B**). Effects of consecutive LH administration on the cellular aging of endometrial stem cells in vivo were determined by measuring SA-β-Gal) activities (**C**). Effects of LH administration on the in vivo mRNA expression levels of several cellular aging genes (*p16* ^*INK4a*^, *p18* ^*INK4c*^, *p21* ^*Cip1*^, and *IL-6*) were also evaluated by performing qPCR (**D**). Suppressive effects of LH on in vivo migration potential of endometrial stem cells were then assessed using transwell assays (**E**) and western blotting for MMP-2 and MMP-9 (**F**). After 2 weeks of differentiation, the suppressive effects of LH on in vivo differentiation abilities of endometrial stem cells into adipocytes (**G**) and osteoblasts **(H**) were evaluated by performing oil red O staining and alizarin red S staining, respectively. Suppressive effects of LH administration on the in vivo expressions of various multipotent capacity-related factors (*C-MYC*, *KLF4*, *NANOG*, *OCT4*, and *SOX2*) were evaluated by performing qPCR (**I**). Uterine endometrial tissue samples from the control or LH-administrated mice were obtained and then fixed in 10% formalin solution for 72 h. Histological examination indicated that the endometrial functional layers of uterine samples were remarkably decreased by consecutive LH administration in vivo (**J**). β-actin was used as an internal control to normalize protein expression. PPIA was used as an internal control to normalize mRNA expression for qPCR analysis. All experiments were performed in triplicates. Data are presented as mean ± standard deviation (SD). *, *p* < 0.05; **, *p* < 0.005; and ***, *p* < 0.001 (two-sample t-test).

Our in vitro results suggested that consecutive LH administration to achieve multiple ovulations during IVF therapy might suppress the tissue regeneration-associated functions in the endometrial stem cells in vivo. Therefore, we administered LH (1 µM per mouse) intraperitoneally to mice for seven consecutive days (seven times) to mimic LH-based multiple ovulation protocols during IVF therapy. Tissue-resident stem cells were successfully isolated from the endometrial tissues of mice (Fig. 8A). Consistent with our in vitro results, consecutive LH administration significantly reduced the self-renewal ability of the tissue-resident endometrial stem cells (Fig. 8B). Exogenous LH administration significantly elevated SA-β-Gal activity (Fig. 8C) and the expression levels of various cellular senescence (aging)-associated genes, such as *IL-6*, *p16*^*INK4A*^, *p18*, and *p21*^*CIP1*^ (Fig. 8D). Transwell assays (Fig. 8E) and western blotting for *MMP-2* and *MMP-9* (Fig. 8F) also demonstrated the suppressive effects of LH on the in vivo migration potential of the endometrial stem cells. In addition, LH remarkably inhibited endometrial stem cell capacity to differentiate into adipocytes (Fig. 8G) and osteoblasts (Fig. 8H). Consecutive LH administration significantly decreased the in vivo mRNA expression levels of pluripotency/stemness-related genes, such as *C-MYC*, *KLF4*, *OCT4*, and *SOX2* in vivo (Fig. 8I). We further investigated whether consecutive LH administration could inhibit the regeneration of the injured endometrial tissue, which is mainly supported by the tissue-resident stem cells. The histological examination of the endometrial tissues showed that the functional layer thickness was remarkably reduced following consecutive LH administration (Fig. 8J). We further examined whether consecutive LH administration inhibited tissue regeneration-associated functions in other stem cell types, such as adipose tissue (Suppl. Fig. 4A) and bone marrow-derived stem cells (Suppl. Fig. 5A). Consistent with the results of the endometrial stem cells, LH administration significantly reduced self-renewal (Suppl. Figs. 4B and 5B), migratory capacity (Suppl. Figs. 4C, D and 5C, D), and differentiation potential (Suppl. Figs. 4E, F and 5E, F) of both the stem cell types. Exogenous LH exposure significantly elevated the SA-β-Gal activity (Suppl. Figs. 4G and 5G) and the expression levels of various cellular senescence (aging)-associated genes (Suppl. Figs. 4H and 5H). In addition, the mRNA expression levels of pluripotency/stemness–related factors, such as *C-MYC*, *KLF4*, *OCT4*, and *SOX2*, were markedly reduced following LH administration in vivo (Suppl. Figs. 4I and 5I) in the adipose tissue- and bone marrow-derived stem cells.

## Discussion

Extensive research previously conducted on various key signaling components and controlling factors that may profoundly affect the tissue regeneration-associated functions in the endometrial stem cells can provide novel insights into previously unidentified causes of endometrial factor-related infertility or recurrent pregnancy loss. Although there are various hormones and chemokines whose primary functions in endometrial stem cells remain poorly defined, significant attention has recently been paid to the adverse effects of exogenous LH administration during IVF therapy, owing to the low fertility rates of LH-based multiple ovulation strategies. At the level of the hypothalamic-pituitary-ovarian axis, LH administration promotes estrogen production, which in turn triggers multiple ovulations and subsequently leads to enhanced pregnancy outcomes [20, 48, 49]. However, a relatively low fertility rate (only between 20% and 35%) is one of the most challenging aspects of the current LH-based multiple ovulation strategy. Moreover, one of the key factors to be considered for women with unexplained repeated miscarriage or recurrent embryo implantation failure in endometrial receptivity, which is an important rate-limiting step in the success of pregnancy in fertility treatment [50, 51]. Until recently, LH-induced effects on pregnancy outcomes have been primarily regarded as indirect (secondary) impacts brought about by LH-mediated estrogen secretion through the pituitary-gonadal axis. Importantly, in addition to their well-known roles in secreting estrogen, LH ligands and their cognate receptors have been detected in several extra ovarian reproductive organs, including the uterine endometrium [17, 52, 53] and placenta [54–56]. Therefore, we postulated that the relatively low pregnancy rates of the multiple LH-based ovulation strategies could be associated with the adverse effects of consecutive LH administration on endometrial receptivity. Additionally, more challenging questions have arisen about the potential direct effects of LH administration on the tissue regeneration-associated functions in the endometrial stem cells, which play a key role in regulating endometrial receptivity. Indeed, endometrial stem cells with high self-renewal ability were not observed in ~40% of endometrial tissues from patients with recurrent miscarriages compared to only 11% of healthy uterine tissues [14]. Until now, the possible direct effects of LH administration on endometrial receptivity and subsequent pregnancy rates remain controversial because of conflicting results from various human studies. To date, some studies have identified some beneficial effects of LH [57–60], whereas other studies have reported its adverse effects [18, 61, 62] on endometrial receptivity. In addition, according to several previous studies, it is known that the pregnancy rates of LH receptor knockout mice were reduced compared to normal mice [63]. Lei et al observed endometrial atrophy with thin all endometrial layers and loss of endometrial glands in LH receptor knockout mice [64]. Estradiol and progesterone levels in serum were significantly suppressed in these mice [64]. However, LH secreted by the pituitary gland mainly acts on the ovaries to produce steroid hormones, thereby stimulating ovarian folliculogenesis, which in turn leads to enhanced pregnancy outcomes. The main target tissue of LH known so far is the ovary, which produces and secretes steroid hormones. In this context, when evaluating the pregnancy rate of LH receptor knockout mice, it is difficult to distinguish whether it is a result of the lack of LH receptor expression in the ovary (LHR knockout-induced downregulation of estrogen production) or the result of lack of LH receptor expression in the endometrium itself.

In contrast to some beneficial effects of LH on HSC models, the present study demonstrates that LH exposure remarkably suppressed several tissue regeneration-associated functions in the endometrial stem cells, such as self-renewal, pluripotency/stemness, migratory potential, and multipotent differentiation ability, both in vitro (Fig. 1A–I) and in vivo (Fig. 8A–J). Similar to our results, Tanaka et al. observed that LHR deficiency is accompanied by increases in the spermatogonial stem cell (SCC) populations and that LH negatively regulates SSC self-renewal by inhibiting the testosterone-induced WNT5A signaling activity [35]. We further studied the underlying molecular mechanisms of the adverse effects of LH on various endometrial stem cell functions. It is known that a number of survival-associated growth factors or chemokines can activate the ERK1/2 [65] and/or PI3K/Akt [66] signaling pathways in many different cell types. Because LH is known to regulate the ERK1/2 and/or PI3K/Akt signaling activities for controlling various cellular functions in multiple cell types [67–69], we investigated whether LH exerts its effect via these signaling pathways through its cognate receptor in endometrial stem cells. LH significantly inhibited both signaling pathways (Fig. 4B, C), and LH-induced suppression of FAK/ERK1/2 or PI3K/Akt signaling was clearly abolished by its receptor knockdown (Fig. 4D, E). Consistently, the harmful effects induced by LH on the tissue regeneration-associated functions were clearly alleviated by SC79 (Fig. 5A–F) or C6-ceramide (Fig. 6A–F) pretreatment. These results indicate that these two signaling activities might serve as key downstream regulators of LH-mediated adverse effects on the beneficial functions of endometrial stem cells.

Taken together, our findings suggest that in addition to its well-known roles in secreting estrogen through the pituitary-gonadal axis, LH can directly suppress the tissue regeneration-associated functions in the endometrial stem cells by inhibiting the FAK/ERK1/2 and/or PI3K/Akt signaling activities. The present study provides novel insights into the previously unanswered reasons for the low fertility rates in multiple LH-based ovulation strategies.

## Materials and methods

### Isolation and culture of human endometrial stem cells from uterine tissues

Human endometrial stem cells were obtained from endometrial tissues of uterine fibroid patients with written informed consent from the patients and approval of the Gachon University Institutional Review Board (IRB No: GAIRB2018-134). We have isolated human endometrial cells from normal areas excluding cancer lesions of pre-menopausal Korean women, undergoing hysterectomy for treatment of uterine fibroids. There is therefore no connection between the origins of endometrial cells and certain pathological conditions. Endometrial tissues used in this experiment were from patients who had not taken any hormone therapy in the previous three months. Reproductive hormones such as estrogen and progesterone, which are dynamically changed during each menstrual cycle, may profoundly affect various physiological characteristics and functions of endometrial stem cells. However, unfortunately, there is no information available for the accurate menstrual stage of endometrial tissue donors. To isolate human endometrial stem cells, endometrial tissue from women undergoing hysterectomy for treatment of uterine fibroids was minced into small pieces, and the small pieces were then digested in DMEM containing 10% FBS and 250 U/ml type I collagenase for 5 h at 37 °C in a rotating shaker. The digestion mixture was then filtered through a 40 µm cell strainer to separate spindle-shaped endometrial stem cells from epithelial gland fragments and undigested tissue. Endometrial stem cells in this suspension were isolated from other cell types by centrifuging for 20 min at 1200 *g* on a single-density Percoll layer. The cells were washed twice in PBS. Endometrial stromal cells were removed from the endometrial cells that were passed through the cell strainer by quick attachment (within 30 min) to a cell culture dish. Unattached endometrial stem cells were harvested, and then cultured in growth media consisted of various growth factors including IGF, VEGF, EGF, basic FGF, hydrocortisone, ascorbic acid, heparin and 10% FBS (Gibco BRL) at 37°C in humidified atmosphere of 5% CO_2_ in the air. After 3 days, colony forming cells were isolated with cloning rings (Sigma-Aldrich). The characteristic information of established endometrial stem cells was summarized in Supple. Table 1. In addition, the specific clinical information of three uterine fibroid patients from whom endometrial stem cells were established and then used in this study was also summarized in Supple. Table. 2.

### Isolation and culture of mouse uterine tissue-derived stem cells

The isolation of mouse uterine tissue-derived stem cells was approved and conducted in accordance with the Institutional Animal Care and Use Committee (IACUC) (LCDI-2020-0006) of the Lee Gil Ya Cancer and Diabetes Institute of Gachon University. Uterine tissue was minced into small pieces, and then the small pieces were digested in DMEM containing 10% FBS and 250 U/ml type I collagenase for 5 h at 37°C. The digestion mixture was then filtered through a 40 µm cell strainer. The endometrial cells were cultured in StemPro® MSC SFM CTS™ (GIBCO, Cat No.: A1033201) at 37 °C, under 5% CO_2_ in air condition. The culture medium was changed every 2 or 3 days.

### Cell proliferation assay

An MTT assay was used to determine the antiproliferative capacity of LH (Cloud-clone, Cat. No.: RPA441Hu01) treatment. Briefly, cells (1 × 10^4^ cells/well) were seeded in 48-well plates in EBM-2 medium (Lonza) supplemented with EGM-2. After 24 h of incubation, the plates were washed with PBS, and the cells were then treated with LH or vehicle for 72 h in serum and supplement-free EBM-2 medium. Thereafter, 50 µl of MTT solution (Sigma–Aldrich, M5655, 5 mg/ml in PBS) was added to each well, and the cells were incubated at 37 °C for another 3 h. Cell viability was assessed by measuring the absorbance at 570 nm using a VersaMax microplate reader. To investigate whether Akt and ERK1/2 signaling activities could mediate LH-induced inhibitory effects on tissue regeneration-associated functions, endometrial stem cells were pretreated with or without Akt activator SC79 (EMD millipore corp, 123871) or ERK1/2 activator ceramide C6 (Santa cruz, sc-3527).

### Senescence-associated beta-galactosidase (SA β-gal) staining

SA β-gal staining was performed as previously described [70]. Endometrial stem cells were seeded in 6-well plates at a density of 1 × 10^5^ cells/well. The cells were incubated for 3 days to the appropriate confluence. The cells were then washed twice with PBS and fixed with 0.5% glutaraldehyde in PBS for 5 min. The cells were then washed with PBS containing 1 mM MgCl_2_ and stained with X-gal solution [1 mg/ml X-gal, 0.12 mM K_3_Fe(CN)_6_, 1 mM MgCl_2_ in PBS (pH 6.0)] overnight at 37 °C.

### In vitro cell migration assay

Cells were plated at 1 × 10^5^ cells/well in 200 μL of culture medium in the upper chambers of Transwell permeable supports (Corning Inc., Corning, NY, USA) to track the migration of cells. The Transwell chambers had 6.5-mm-diameter polycarbonate membranes with an 8.0 μm pore size in a 24-well plate format. Noninvaded cells on the upper surface of each membrane were removed by scrubbing with a Kimwipe. Migrated cells on the lower surface of each membrane were fixed with 4% paraformaldehyde for 5 min and stained with hematoxylin for 15 min. Later, the number of migrated cells was counted in three randomly selected fields of view for each well under a light microscope at 50X magnification. To calculate the migration index, the number of cells that migrated in response to LH treatment was divided by the number of spontaneously migrating cells.

### Real-time PCR

Total RNA was extracted using TRIzol reagent (Invitrogen) according to the manufacturer’s protocol. RNA purity was verified by measuring the 260/280 absorbance ratio. First-strand cDNA was synthesized from 1 μg of total RNA using SuperScript II (Invitrogen), and one-tenth of the cDNA was added to each PCR mixture containing Express SYBR-Green qPCR Supermix (BioPrince, Seoul, South Korea). Real-time PCR was performed using a Rotor-Gene Q thermocycler (Qiagen). PCR was performed with 40 cycles of amplification with denaturation at 95 °C for 20 sec, annealing at 60 °C for 20 sec, and extension at 72 °C for 25 sec. The relative mRNA expression of the selected genes was normalized to that of PPIA and quantified using the ΔΔCT method. The sequences of the PCR primers are listed in Table 1.

**Table 1:** Primer sequences for quantitative RT-PCR.

Gene	Gene bank no.	Direction	Primer sequence
Human PPIA	NM_021130	F	TGCCATCGCCAAGGAGTAG
		R	TGCACAGACGGTCACTCAAA
Human IL6	NM_000600	F	GGTACATCCTCGACGGCATCT
		R	GTGCCTCTTTGCTGCTTTCAC
Human P16	NM_000077	F	CTACTGAGGAGCCAGCGTCT
		R	CTGCCCATCATCATGACCT
Human P18	NM_001262	F	TGGGTCTTCCGCAAGAACTC
		R	TGGCAGCCAAGTGCAAGGGC
Human P21	NM_000389	F	ACAGCAGAGGAAGACCATGTGGACC
		R	CGTTTTCGACCCTGAGAGTCTCCAG
Human C-MYC	NM_002467	F	AAAGGCCCCCAAGGTAGTTA
		R	GCACAAGAGTTCCGTAGCTG
Human KLF4	NM_001314052	F	GAACTGACCAGGCACTACCG
		R	TTCTGGCAGTGTGGGTCATA
Human NANOG	NM_024865	F	TGGGATTTACAGGCGTGAGC
		R	AAGCAAAGCCTCCCAATCCC
Human OCT4	NM_002701	F	AGCCCTCATTTCACCAGGCC
		R	TGGGACTCCTCCGGGTTTTG
Human SOX2	NM_003106	F	AAATGGGAGGGGTGCAAAAGAGGAG
		R	CAGCTGTCATTTGCTGTGGGTGATG
Human LHR	NM_000233	F	ACCTCCCTGTCAAAGTGATCC
		R	AGGTTGTCAAAGGCATTAGCTTC
Mouse HPRT	NM_013556	F	GCCTAAGATGAGCGCAAGTTG
		R	TACTAGGCAGATGGCCACAGG
Mouse C-MYC	NM_010849	F	CGCACACACAACGTCTTGGA
		R	AGGATGTAGGCGGTGGCTTT
Mouse KLF4	NM_010637	F	GGTGCAGCTTGCAGCAGTAA
		R	AAAGTCTAGGTCCAGGAGGT
Mouse OCT4	NM_013633	F	GCATTCAAACTGAGGCACCA
		R	AGCTTCTTTCCCCATCCCA
Mouse SOX2	NM_011443	F	GAAGCGTGTACTTATCCTTCTTCAT
		R	GAGTGGAAACTTTTGTCCGAGA
Mouse IL6	NM_031168	F	TAGTCCTTCCTACCCCAATTTCC
		R	TTGGTCCTTAGCCACTCCTTC
Mouse P16	NM_001040654	F	TTGAGCAGAAGAGCTGCTACGT
		R	CGTACCCCGATTCAGGTGAT
Mouse P18	NM_001301368	F	GAACCATAAGGGGGACACCG
		R	CCATTTGCCTCCATCAGGCT
Mouse P21	NM_007669	F	GGTTCCTTGCCACTTCTT
		R	GAGTCGGGATATTACGGTTG

### Protein isolation and western blot analysis

Protein expression levels were determined by western blot analysis as previously described [71]. Cells were lysed in a buffer containing 50 mM Tris, 5 mM EDTA, 150 mM NaCl, 1 mM DTT, 0.01% NP 40, and 0.2 mM PMSF. The protein concentrations in the total cell lysates were measured by using bovine serum albumin as the standard. Samples containing equal amounts of protein were separated via sodium dodecyl sulfate-polyacrylamide gel electrophoresis (SDS–PAGE), and proteins were then transferred onto nitrocellulose membranes (Bio–Rad Laboratories). Membranes were blocked with 5% skim milk in Tris-buffered saline containing Tween 20 at RT. Then, the membranes were incubated with primary antibodies against MMP-2 (Cell Signaling #4022), MMP-9 (Cell Signaling #13667), total PI3K (Cell Signaling #4292), phospho-PI3K (Cell Signaling #4228), total Akt (Cell Signaling #4491), phospho-Akt (Cell Signaling #4060), total-ERK1/2 (Cell Signaling #9012), phospho-ERK1/2 (Cell Signaling #9101), total FAK (Santa Cruz, sc-558), phospho-FAK (Santa Cruz, sc-11765), or β-actin (Abcam, ab189073) overnight at 4 °C and then with HRP-conjugated goat anti-rabbit IgG (BD Pharmingen, San Diego, CA, USA, 554021) and goat anti-mouse IgG (BD Pharmingen, 554002) secondary antibodies for 60 min at RT. Antibody-bound proteins were detected using ECL reagents.

### Adipogenic differentiation

Endometrial stem cells were incubated in low-glucose DMEM supplemented with 500 µM methylxanthine, 5 µg/mL insulin, and 10% FBS and were treated with or without LH treatment. Endometrial stem cells were grown for 3 weeks, with medium replacement twice a week. Lipid droplet formation was confirmed by Oil Red O staining. Relative quantification of lipid droplet formation was determined by measuring the absorbance at 500 nm.

### Osteogenic differentiation

Endometrial stem cells were incubated in high-glucose DMEM supplemented with 0.1 µM dexamethasone, 10 mM β-glycerophosphate, 50 µM ascorbate, and 10% FBS and were treated with or without LH treatment. Endometrial stem cells were grown for 3 weeks, with medium replacement twice a week. Differentiated cells were stained with Alizarin Red S to detect de novo formation of bone matrix. Alizarin red S staining in the samples was quantified by measuring the optical density (OD) of the solution at 570 nm.

### Analysis of mitochondrial respiration and glycolytic capacity

With a Seahorse XF analyzer (Seahorse Bioscience, North Billerica, MA), mitochondrial oxidative phosphorylation and glycolytic flux can be analyzed in real-time by measuring the OCR and ECAR of cells as they respond to substrates and metabolism-inhibiting agents according to the manufacturer’s instructions [72]. The ATP synthase inhibitor oligomycin (a complex V blocker) is added to inhibit ATP-coupled respiration. FCCP (a mitochondrial uncoupler) is added to collapse the mitochondrial membrane potential (Δψm). Rotenone (an inhibitor of complex I in the electron transport chain) and antimycin A (an inhibitor of complex III in the electron transport chain) are added to block mitochondrial respiration completely. To measure real-time glycolytic rates, the Seahorse XF glycolytic rate assay utilizes both extracellular acidification rate (ECAR) and OCR measurements to evaluate the glycolytic proton efflux rate (glycoPER) of the cells; in this assay, cells are incubated in glucose-free medium to which rotenone, antimycin A, and finally 2-deoxyglucose (2-DG, a glycolysis inhibitor) are sequentially added. The OCR and ECAR were described as absolute rates (pmoles/min for OCR and mpH/min for ECAR) and normalized to the cell number as a percentage of the baseline oxygen consumption.

### LH receptor (LHR) knockdown

Small hairpin RNA (shRNA; accession no. NM_000233) targeting LH receptor and scrambled shRNA (Sigma, SHC001) were purchased from Bioneer (Daejeon, South Korea). For efficient LHR transfection, reverse transfection was performed using Lipofectamine 2000 (Invitrogen) according to the manufacturer’s protocol. Briefly, shRNA targeting LHR (3 μg/ml) was mixed with 3 μl of the transfection reagent Lipofectamine 2000 in Gibco Opti-MEM medium without FBS and antibiotics. Five hours before transfection, the Opti-MEM was replaced with fresh EGM-2 supplemented with 10% FBS. We chose the LHR shRNA that was most effective at the mRNA level from three shRNAs designed from the target sequence and analyzed by qRT–PCR.

### Analysis of the GEO database

Gene Expression Omnibus (GEO) (https://www.ncbi.nlm.nih.gov/geo/) is a freely distributed database of high-throughput gene expression data generated by genome hybridization array, chip sequencing, and DNA microarray analyses [73, 74]. Researchers provide their experimental results in four categories: experimental designs, samples, platforms, and raw data. Clinical or experimental samples within each dataset are further organized based on various experimental subgroups, such as treatment, physiologic condition, and disease state. These categorized biological data are presented as a “GEO profile”, which includes the dataset title, gene annotation, a chart depicting the expression levels, and the rank for that gene across each sample [75]. The expression profiles of Akt2, PI3K (Pik3r2), ERK (EphB3), FAK (PTK2b), IGFBP3, IGFBP4, IGFBP6, or NT-3 in various physiological conditions were analyzed according to previously established procedures [75].

### Growth factor antibody array assay

The assay was performed following the manufacturer’s protocol (Abnova AA0089). Briefly, were incubated with antibody-spotted membranes were incubated with LH- or vehicle-treated protein samples overnight at 4 °C. After washing three times with wash buffer, the membranes were incubated with biotin-conjugated anti-cytokine antibodies overnight at 4 °C. The membranes were then washed three times and incubated with HRP-conjugated streptavidin. Chemiluminescence was used to detect signals from the growth factor antibodies spotted on the nitrocellulose membrane.

### Evaluation of the effects of LH treatment in an animal model

All of the animal experiments were approved and conducted in accordance with the Institutional Animal Care and Use Committee (IACUC) (LCDI-2020-0006) of Gachon University. The ICR mice (Female, 8-week-old) were randomly divided into control and LH treatment (1 µg/mouse for 7 consecutive days intravenously) groups. The mice were anesthetized and exsanguinated by cardiac puncture, and then stem cells were isolated from uterine, adipose, and bone marrow tissues, respectively. For further experiments, isolated stem cells from these tissues were cultured and expanded in vitro with continuous exposure to LH (25 nM/ml) to properly mimic physiological conditions of LH exposure in vivo.

### Statistical analysis

All in vivo and in vitro data were presented as mean ± S.D. of three independent experimental repeats. All statistical data were analyzed with GraphPad Prism 5.0 (GraphPad Software, San Diego, CA) and evaluated using two-tailed Student’s *t* tests. Values of *P* < 0.05 were considered to indicate statistical significance. The variance between the groups was not significant. All the samples were not excluded, and investigators were not blinded to the group allocation.

## Supplementary information

Author-contribution-form

aj-checklist.

Supplementary figures and legends

Original Data File

## Data Availability

All data sets are included in this published article and its supplementary information. Additional data are available from the corresponding author on reasonable request.
