# Supplementary material for: Novel roles of luteinizing hormone (LH) in tissue regeneration-associated functions in endometrial stem cells
Source: Cell Death Dis. 2022 Jul 13;13(7):605. doi: 10.1038/s41419-022-05054-7 (PMC9279474; doi:10.1038/s41419-022-05054-7)
Supplement: Supplementary file 1 — Author-contribution-form [file 41419_2022_5054_MOESM1_ESM.pdf]

DECLARATION OF CONTRIBUTIONS TO ARTICLE

ADMC

Manuscript Number: CDDIS-21-4444RRR

Journal Name: *Cell Death & Disease* (the 'Journal')

Proposed Title of the Contribution:

Novel roles of luteinizing hormone (LH) in tissue regeneration-associated [illegible] (the 'Contribution)

Author(s):

Se-Ra Park, Seong-Kwan Kim, Soo-Rim Kim, Jeong-Ran Park, Soyi Lim, In-Sun [illegible] (the 'Authors')

For all *CDDis* articles, each person named as an author in the published version must be able to show he or she has contributed substantially to the article.

Authorship credit should be based on 1) substantial contributions to conception and design, acquisition of data, or analysis and interpretation of data; 2) drafting the article or revising it critically for important intellectual content; and 3) final approval of the version to be published. Authors should meet conditions 1, 2 and 3.

Any person who cannot be shown to have made a substantial contribution to the article cannot be listed as an author in the final version. The name of any person who is deemed to have made a minor contribution can, however, appear in the Acknowledgments section of the article.

Please complete the table below to indicate the contributions of all named authors to the manuscript.

Author Full Name:	Specification of Contribution to the Manuscript:
Se-Ra Park	designed and performed experiments, analyzed data and wrote the paper
Seong-Kwan Kim	designed and performed experiments, analyzed data and wrote the paper
Soo-Rim Kim	designed and performed experiments, analyzed data and wrote the paper
Jeong-Ran Park	designed and performed experiments, analyzed data and wrote the paper
Soyi Lim	designed and performed experiments, analyzed data and wrote the paper
In-Sun Hong	designed experiments, analyzed data and wrote the paper

Please complete the table below to indicate the contributions of all named authors to the figures.

Figure 1:

S.R.P., S.K.K., S.R.K.,J.R.P., and S.L.,designed and performed experiments, and analyzed data. I.S.H. and H.Y.L. designed experiments and analyzed data.

Figure 2:

S.R.P., S.K.K., S.R.K., J.R.P., and S.L.,designed and performed experiments, and analyzed data. I.S.H. and H.Y.L. designed experiments and analyzed data.

Figure 3:

S.R.P., S.K.K., S.R.K., J.R.P., and S.L.,designed and performed experiments, and analyzed data. I.S.H. and H.Y.L. designed experiments and analyzed data.

Figure 4:

S.R.P., S.K.K., S.R.K., J.R.P., and S.L.,designed and performed experiments, and analyzed data. I.S.H. and H.Y.L. designed experiments and analyzed data.

Figure 5:

S.R.P., S.K.K., S.R.K., J.R.P., and S.L.,designed and performed experiments, and analyzed data. I.S.H. and H.Y.L. designed experiments and analyzed data.

Figure 6:

S.R.P., S.K.K., S.R.K., J.R.P., and S.L.,designed and performed experiments, and analyzed data. I.S.H. and H.Y.L. designed experiments and analyzed data.

Signed for and on behalf of the Author(s):	Print Name:	Date:
	In-Sun Hong	JUNE.23.2022
