# Supplementary material for: Novel roles of luteinizing hormone (LH) in tissue regeneration-associated functions in endometrial stem cells
Source: Cell Death Dis. 2022 Jul 13;13(7):605. doi: 10.1038/s41419-022-05054-7 (PMC9279474; doi:10.1038/s41419-022-05054-7)
Supplement: Supplementary file 3 — Supplementary figures and legends [file 41419_2022_5054_MOESM3_ESM.pdf]

Supplementary figures and legends

Supplementary figure 1

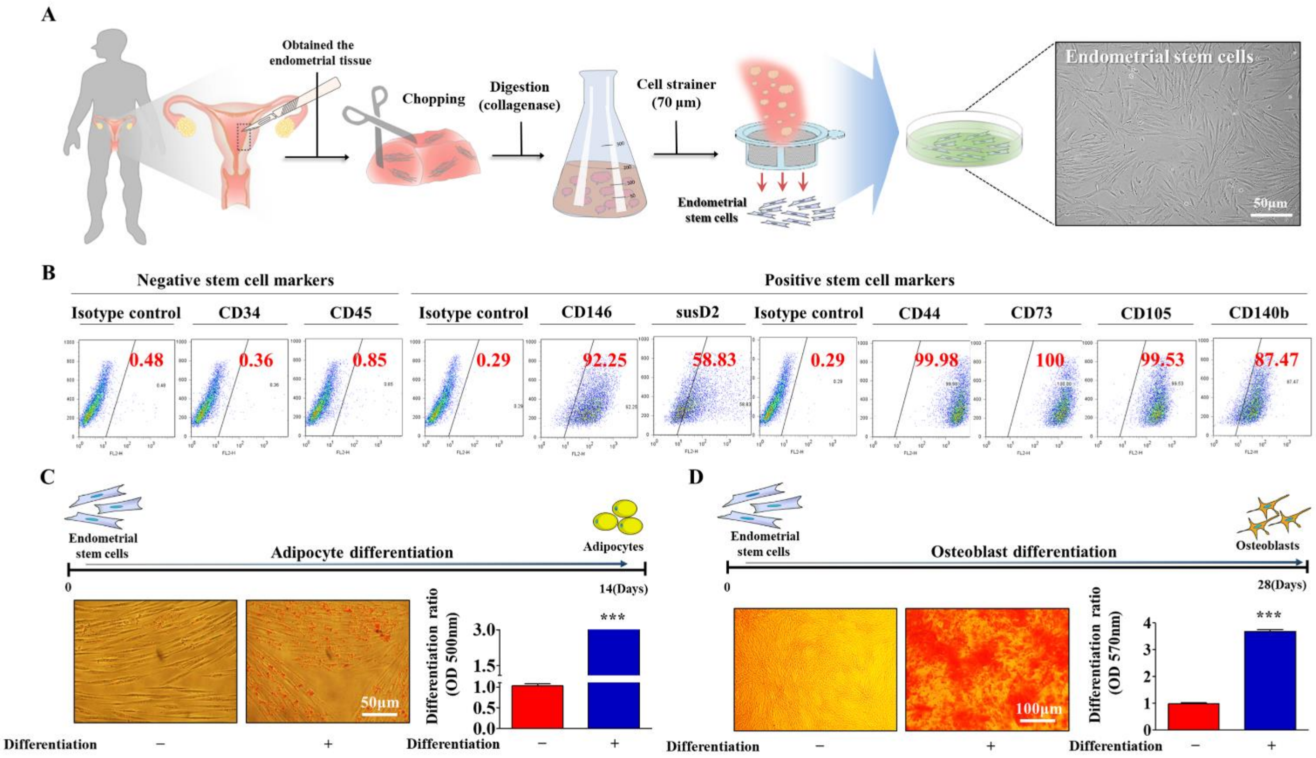

Suppl. Fig. 1 Isolation and characterization of human endometrial stem cells from endometrial tissues. Endometrial tissue was minced into small pieces, and then the small pieces were digested with type I collagenase. Isolated human endometrial stem cells were observed under an inverted phase-contrast microscope to assess their morphological characterization **(a)**. The isolated endometrial stem cells were analyzed using flow cytometry with various antibodies for identified stem cell markers (CD44, CD73, CD105, CD140b, CD146, and susD2) and several hematopoietic markers (CD34 and CD45) **(b)**. Their ability to differentiate into adipocytes **(c)** and osteoblasts **(d)** was analyzed using oil red O and alizarin red S staining, respectively. The relative quantification of calcium deposition and lipid droplet (LD) secretion from differencing cells were assessed by measuring the absorbance of the solubilized cells at 500 nm and 570 nm, respectively. All experiments were performed in triplicates, and the data has been presented as mean ± standard deviation (SD). $*p < 0.05$, $**p < 0.005$, and $***p < 0.001$ (two-sample t-test).

Supplementary figure 2

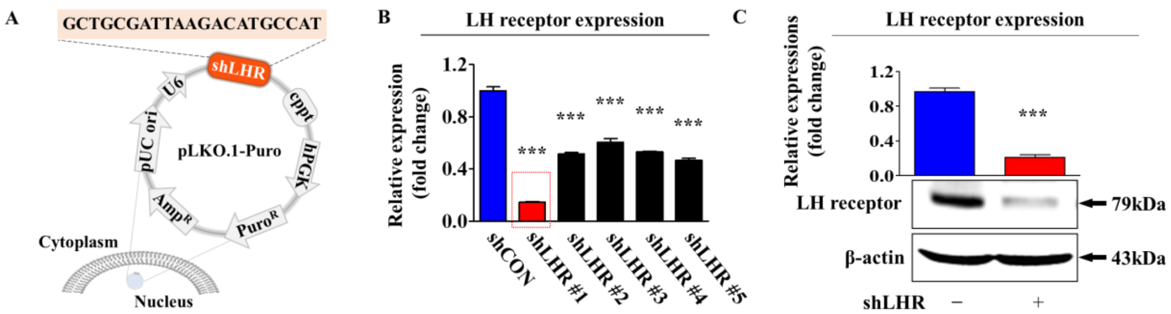

Suppl. Fig. 2. Knockdown efficacy of five different shRNA constructs selectively targeting LH receptor (LHR). Endometrial stem cells were transfected with a non-targeting scramble shRNA or five different shRNA constructs, which selectively target LHR **(A)**. LHR shRNA construct #1, hereafter indicated as LHR shRNA, showed the most effective knockdown efficacy. The knockdown efficacy of LHR was determined by performing real-time PCR and western blotting at mRNA **(B)** and protein **(C)** expression levels. β-actin was used as an internal control to normalize protein expression. PPIA was used as an internal control to normalize mRNA expression for qPCR analysis. All experiments were performed in triplicates, and the data has been presented as mean ± standard deviation (SD). *$p < 0.05$, **$p < 0.005$, and ***$p < 0.001$ (two-sample t-test).

Supplementary figure 3

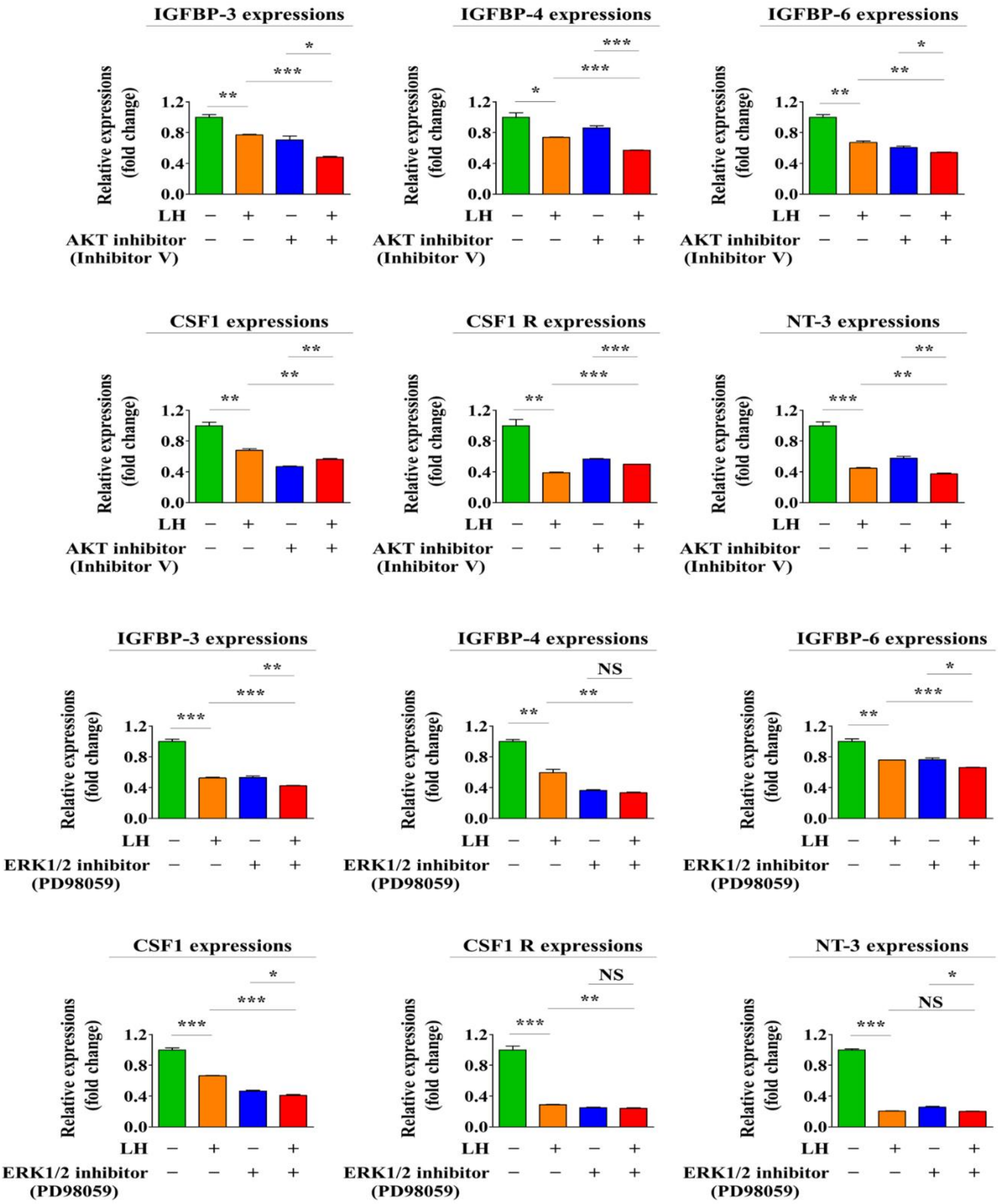

Suppl. Fig. 3. Expression levels of six prominent factors in response LH treatment. Inhibitory effects of LH treatment on mRNA levels of six prominent factors [insulin-like growth factor binding protein (IGFBP) 3, 4, 6, colony stimulating factor 1 (CSF1), colony stimulating factor 1 receptor

(CSF1R), and neurotrophin 3 (NT3)] were analyzed by real-time PCR. PPIA was used as a housekeeping gene for real-time PCR analysis. All experiments were performed in triplicates. Data are presented as mean ± standard deviation (SD). *, $p < 0.05$; **, $p < 0.005$; and ***, $p < 0.001$ (two-sample t-test).

Supplementary figure 4

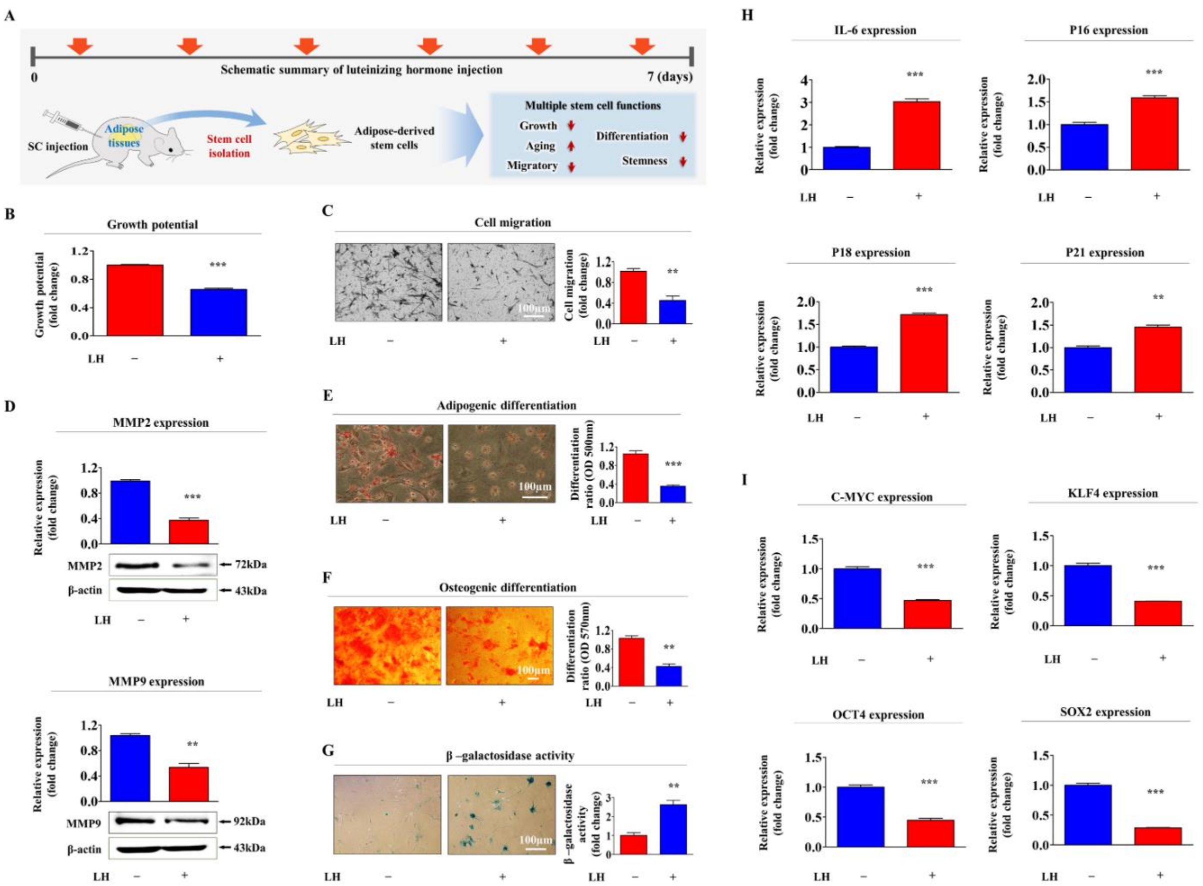

Suppl. Fig. 4. Consecutive LH administration inhibits tissue regeneration-associated functions in adipose tissue-derived stem cells *in vivo*. A schematic summary of the overall *in vivo* experimental procedure is described in the Materials and Methods' section **(A)**. Each mouse was administered LH (1 μM/mouse, daily for 7 consecutive days) via intravenous injection through the tail vein. Adipose tissue-derived stem cells were successfully obtained from mouse adipose tissues using our previously established primary culture protocol. During *in vitro* expansion, isolated cells were cultured either under continuous LH (25 nM) exposure or non-exposure conditions to mimic the *in vivo* environment of consecutive LH administration. Their effects on the self-renewal ability of adipose tissue-derived stem cells were determined using MTT-based assays. Cell growth rates were estimated as the relative viability of the LH-treated groups as a percentage (%) of the vehicle-treated groups **(B)**. Effects of consecutive LH administration on the *in vivo* cellular aging of adipose tissue-

derived stem cells were determined by measuring SA-β-Gal activity **(C)**. The effects of LH administration on the *in vivo* mRNA expression levels of several cellular aging genes (*p16* ^{INK4a}, *p18* ^{INK4c}, *p21* ^{Cip1}, and *IL-6)* were also evaluated using qPCR **(D)**. Suppressive effects of LH on the *in vivo* migration potential of adipose tissue-derived stem cells were assessed using transwell assays **(E)** and western blotting for MMP-2 and MMP-9 **(F)**. After 2 weeks of differentiation, the suppressive effects of LH on the *in vivo* differentiation abilities of adipose tissue-derived stem cells into adipocytes **(G)** and osteoblasts **(H)** were evaluated using Oil Red O staining and Alizarin Red S staining, respectively. Suppressive effects of LH administration on the *in vivo* expression levels of various multipotent capacity-related factors (*C-MYC*, *KLF4*, *NANOG*, *OCT4*, and *SOX2*) were evaluated by performing qPCR **(I)**. β-actin was used as an internal control to normalize protein expression. PPIA was used as an internal control to normalize mRNA expression for qPCR analysis. All experiments were performed in triplicates. Data are presented as mean ± standard deviation (SD). *, $p < 0.05$; **, $p < 0.005$; and ***, $p < 0.001$ (two-sample t-test).

Supplementary figure 5

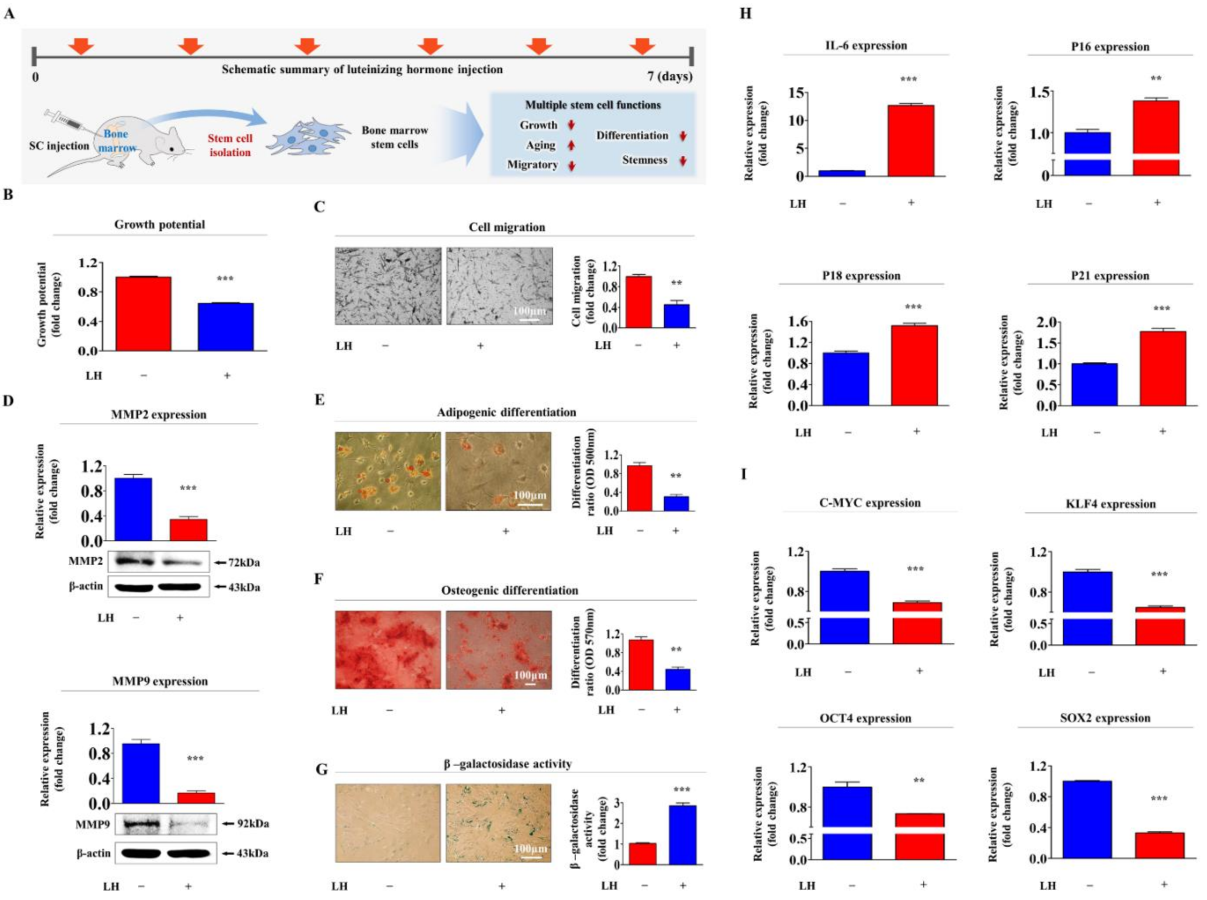

Suppl. Fig. 5. Consecutive LH administration remarkably inhibits tissue regeneration-associated functions in bone marrow-derived stem cells *in vivo*. A schematic summary of the overall *in vivo* experimental procedure is described in the Materials and Methods' section **(A)**. Each mouse was administered LH (1 μM/mouse daily for 7 consecutive days) via intravenous injection through the tail vein. Next, bone marrow-derived stem cells were successfully obtained from the mouse bone marrow using our previously established primary culture protocol. During *in vitro* expansion, isolated cells were cultured either under continuous LH (25 nM) exposure or non-exposure conditions to mimic the *in vivo* environment of consecutive LH administration. Their effects on the self-renewal ability of bone marrow-derived stem cells were determined using MTT-based assays. Cell growth rates were estimated as the relative viability of the LH-treated groups as a percentage (%) of the vehicle-treated groups **(B)**. Effects of consecutive LH administration on the cellular aging of

bone marrow-derived stem cells *in vivo* were determined by measuring SA-β-Gal activity **(C)**. The effects of LH administration on the *in vivo* mRNA expression levels of several cellular aging genes (*p16* ^{INK4a}, *p18* ^{INK4c}, *p21* ^{Cip1}, and *IL-6*) were also evaluated using qPCR **(D)**. Suppressive effects of LH on the *in vivo* migration potential of bone marrow-derived stem cells were assessed using transwell assays **(E)** and western blotting for MMP-2 and MMP-9 **(F)**. After 2 weeks of differentiation, the suppressive effects of LH on the *in vivo* differentiation abilities of bone marrow-derived stem cells into adipocytes **(G)** and osteoblasts **(H)** were evaluated using Oil Red O staining and Alizarin Red S staining, respectively. Suppressive effects of LH administration on the *in vivo* expression levels of various multipotent capacity-related factors (*C-MYC*, *KLF4*, *NANOG*, *OCT4*, and *SOX2*) were evaluated by performing qPCR **(I)**. β-actin was used as an internal control to normalize protein expression. PPIA was used as an internal control to normalize mRNA expression for qPCR analysis. All experiments were performed in triplicates. Data are presented as mean ± standard deviation (SD). *, $p < 0.05$; **, $p < 0.005$; and ***, $p < 0.001$ (two-sample t-test).

Supplementary table 1. The characteristic information of established endometrial stem cells.

<table>
<tr><th colspan="24">Cell line #1 (Patient NO.: 35946553)</th></tr>
<tr><td colspan="24">Isolation date: 2018.08.31</td></tr>
<tr><td colspan="8">Growth potential</td><td colspan="8">Adipogenic differentiation</td><td colspan="8">Osteogenic differentiation</td></tr>
<tr><td colspan="8">OK</td><td colspan="8">OK</td><td colspan="8">OK</td></tr>
<tr><td colspan="24">FACS (%)</td></tr>
<tr><td colspan="3">CD34</td><td colspan="3">CD45</td><td colspan="3">CD44</td><td colspan="3">CD73</td><td colspan="3">CD105</td><td colspan="3">CD146</td><td colspan="3">CD140b</td><td colspan="3">SUSD2</td></tr>
<tr><td colspan="3">0.56</td><td colspan="3">0.77</td><td colspan="3">99.82</td><td colspan="3">99.94</td><td colspan="3">99.51</td><td colspan="3">83.72</td><td colspan="3">99.52</td><td colspan="3">20.88</td></tr>
<tr><td colspan="24">The levels of pluripotency-related genes (fold changes compared to fibroblasts)</td></tr>
<tr><td colspan="6">OCT4</td><td colspan="6">SOX2</td><td colspan="6">NANOG</td><td colspan="6">C-MYC</td></tr>
<tr><td colspan="6">3.74</td><td colspan="6">18.8</td><td colspan="6">240.8</td><td colspan="6">2.53</td></tr>
<tr><th colspan="24">Cell line #2 (Patient NO.: 07939623)</th></tr>
<tr><td colspan="24">Isolation date: 2018.10.8</td></tr>
<tr><td colspan="8">Growth potential</td><td colspan="8">Adipogenic differentiation</td><td colspan="8">Osteogenic differentiation</td></tr>
<tr><td colspan="8">OK</td><td colspan="8">OK</td><td colspan="8">OK</td></tr>
<tr><td colspan="24">FACS (%)</td></tr>
<tr><td colspan="3">CD34</td><td colspan="3">CD45</td><td colspan="3">CD44</td><td colspan="3">CD73</td><td colspan="3">CD105</td><td colspan="3">CD146</td><td colspan="3">CD140b</td><td colspan="3">SUSD2</td></tr>
<tr><td colspan="3">0.23</td><td colspan="3">2.36</td><td colspan="3">99.99</td><td colspan="3">99.96</td><td colspan="3">99.88</td><td colspan="3">65.65</td><td colspan="3">99.95</td><td colspan="3">69.51</td></tr>
<tr><td colspan="24">The levels of pluripotency-related genes (fold changes compared to fibroblasts)</td></tr>
<tr><td colspan="6">OCT4</td><td colspan="6">SOX2</td><td colspan="6">NANOG</td><td colspan="6">C-MYC</td></tr>
<tr><td colspan="6">294.3</td><td colspan="6">5.3</td><td colspan="6">287.3</td><td colspan="6">7.4</td></tr>
<tr><th colspan="24">Cell line #3 (Patient NO.: 23257133)</th></tr>
<tr><td colspan="24">Isolation date: 2018.10.16</td></tr>
<tr><td colspan="8">Growth potential</td><td colspan="8">Adipogenic differentiation</td><td colspan="8">Osteogenic differentiation</td></tr>
<tr><td colspan="8">OK</td><td colspan="8">OK</td><td colspan="8">OK</td></tr>
<tr><td colspan="24">FACS (%)</td></tr>
<tr><td colspan="3">CD34</td><td colspan="3">CD45</td><td colspan="3">CD44</td><td colspan="3">CD73</td><td colspan="3">CD105</td><td colspan="3">CD146</td><td colspan="3">CD140b</td><td colspan="3">SUSD2</td></tr>
<tr><td colspan="3">1.04</td><td colspan="3">8.21</td><td colspan="3">99.95</td><td colspan="3">99.98</td><td colspan="3">99.89</td><td colspan="3">79.52</td><td colspan="3">99.98</td><td colspan="3">76.26</td></tr>
<tr><td colspan="24">The levels of pluripotency-related genes (fold changes compared to fibroblasts)</td></tr>
<tr><td colspan="6">OCT4</td><td colspan="6">SOX2</td><td colspan="6">NANOG</td><td colspan="6">C-MYC</td></tr>
<tr><td colspan="6">397.2</td><td colspan="6">7.4</td><td colspan="6">376.1</td><td colspan="6">9.2</td></tr>
</table>

Supplementary table 2. The clinical information of three uterine fibroid patients from whom endometrial stem cells were established.

	Patient #1	Patient #2	Patient #3
Age (years)	40	48	49
BMI (kg/m^2)	24.64	25.27	18.96
Height (cm)	155	154	164
Weight (kg)	64	60.4	51
DM	N/A	OK	N/A
Menstrual cycle	Less than 28 days	Normal cycle	Normal cycle
