# Supplementary material for: Novel roles of luteinizing hormone (LH) in tissue regeneration-associated functions in endometrial stem cells
Source: Cell Death Dis. 2022 Jul 13;13(7):605. doi: 10.1038/s41419-022-05054-7 (PMC9279474; doi:10.1038/s41419-022-05054-7)

Figure 1F

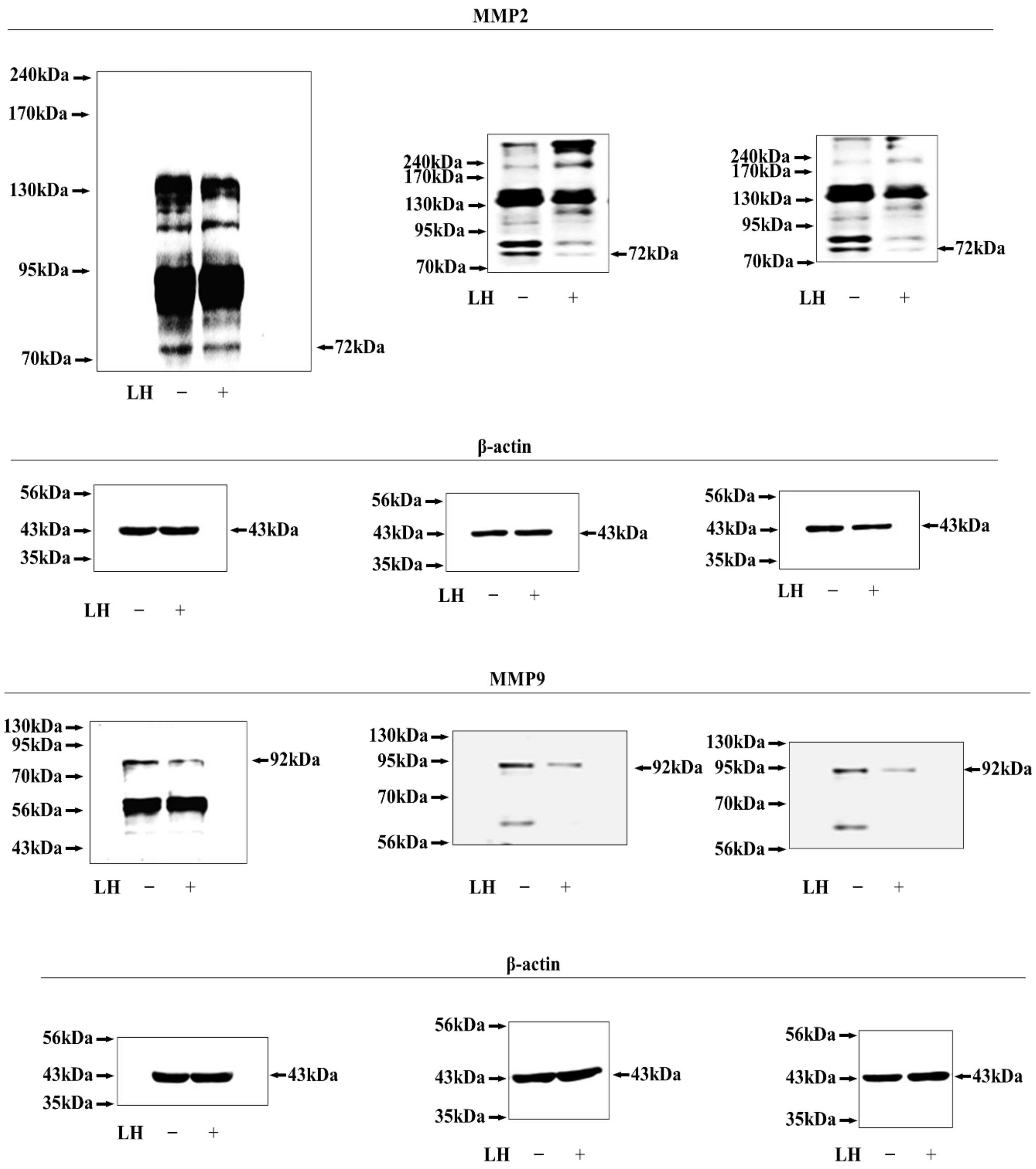
MMP2
240kDa
170kDa
130kDa
95kDa
70kDa
72kDa
LH − +
240kDa
170kDa
130kDa
95kDa
70kDa
72kDa
LH − +
240kDa
170kDa
130kDa
95kDa
70kDa
72kDa
LH − +
β-actin
56kDa
43kDa
35kDa
43kDa
LH − +
56kDa
43kDa
35kDa
43kDa
LH − +
56kDa
43kDa
35kDa
43kDa
LH − +
MMP9
130kDa
95kDa
70kDa
56kDa
43kDa
92kDa
LH − +
130kDa
95kDa
70kDa
56kDa
92kDa
LH − +
130kDa
95kDa
70kDa
56kDa
92kDa
LH − +
β-actin
56kDa
43kDa
35kDa
43kDa
LH − +
56kDa
43kDa
35kDa
43kDa
LH − +
56kDa
43kDa
35kDa
43kDa
LH − +

Figure 3D

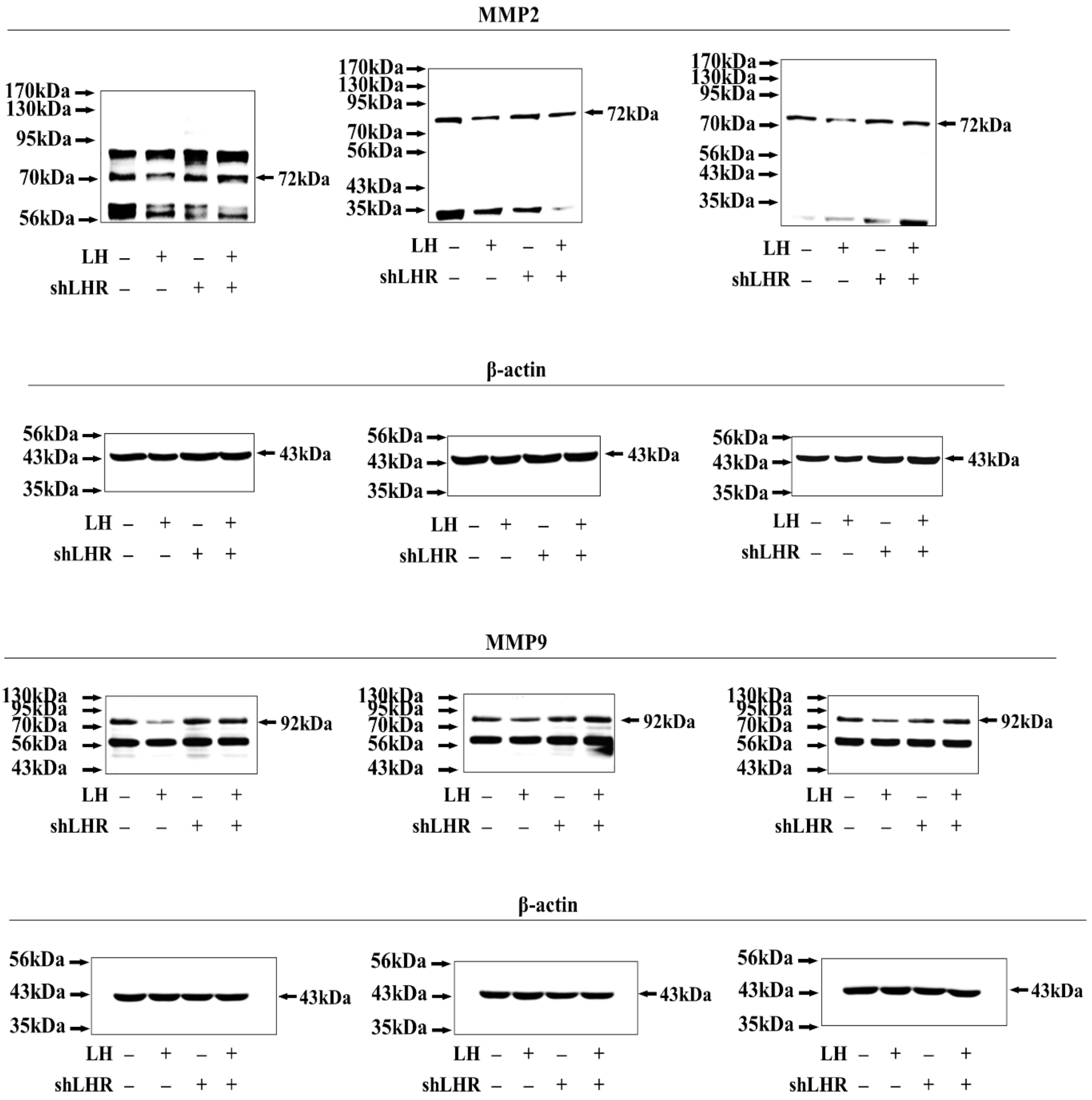

MMP2
170kDa
130kDa
95kDa
70kDa
56kDa
43kDa
35kDa
72kDa
LH – + – +
shLHR – – + +
β-actin
56kDa
43kDa
35kDa
43kDa
LH – + – +
shLHR – – + +
MMP9
130kDa
95kDa
70kDa
56kDa
43kDa
92kDa
LH – + – +
shLHR – – + +
β-actin
56kDa
43kDa
35kDa
43kDa
LH – + – +
shLHR – – + +

Figure 4B

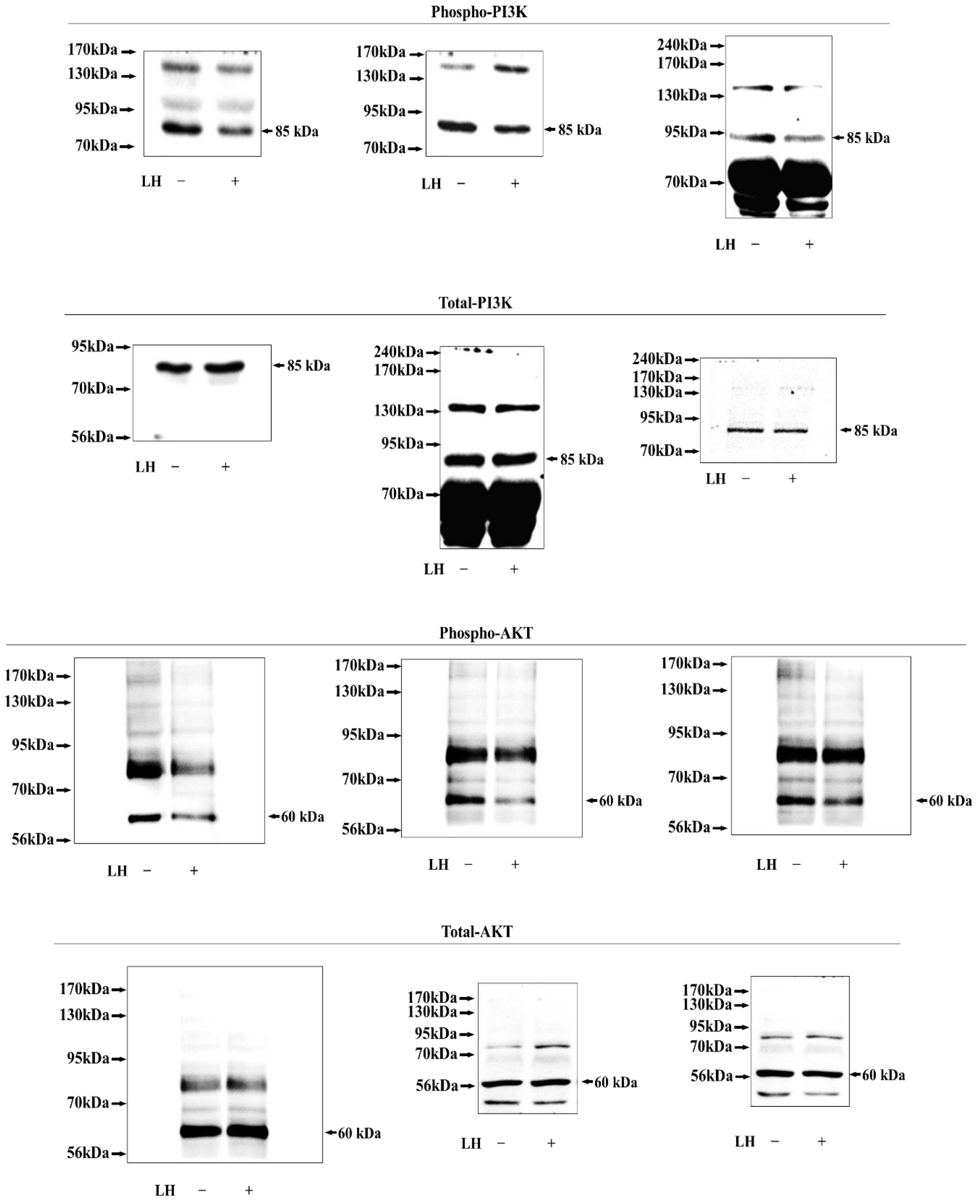
Phospho-PI3K
170kDa
130kDa
95kDa
70kDa
85 kDa
240kDa
LH − +
Total-PI3K
95kDa
70kDa
56kDa
85 kDa
240kDa
170kDa
130kDa
LH − +
Phospho-AKT
170kDa
130kDa
95kDa
70kDa
56kDa
60 kDa
LH − +
Total-AKT
170kDa
130kDa
95kDa
70kDa
56kDa
60 kDa
LH − +

Figure 4C

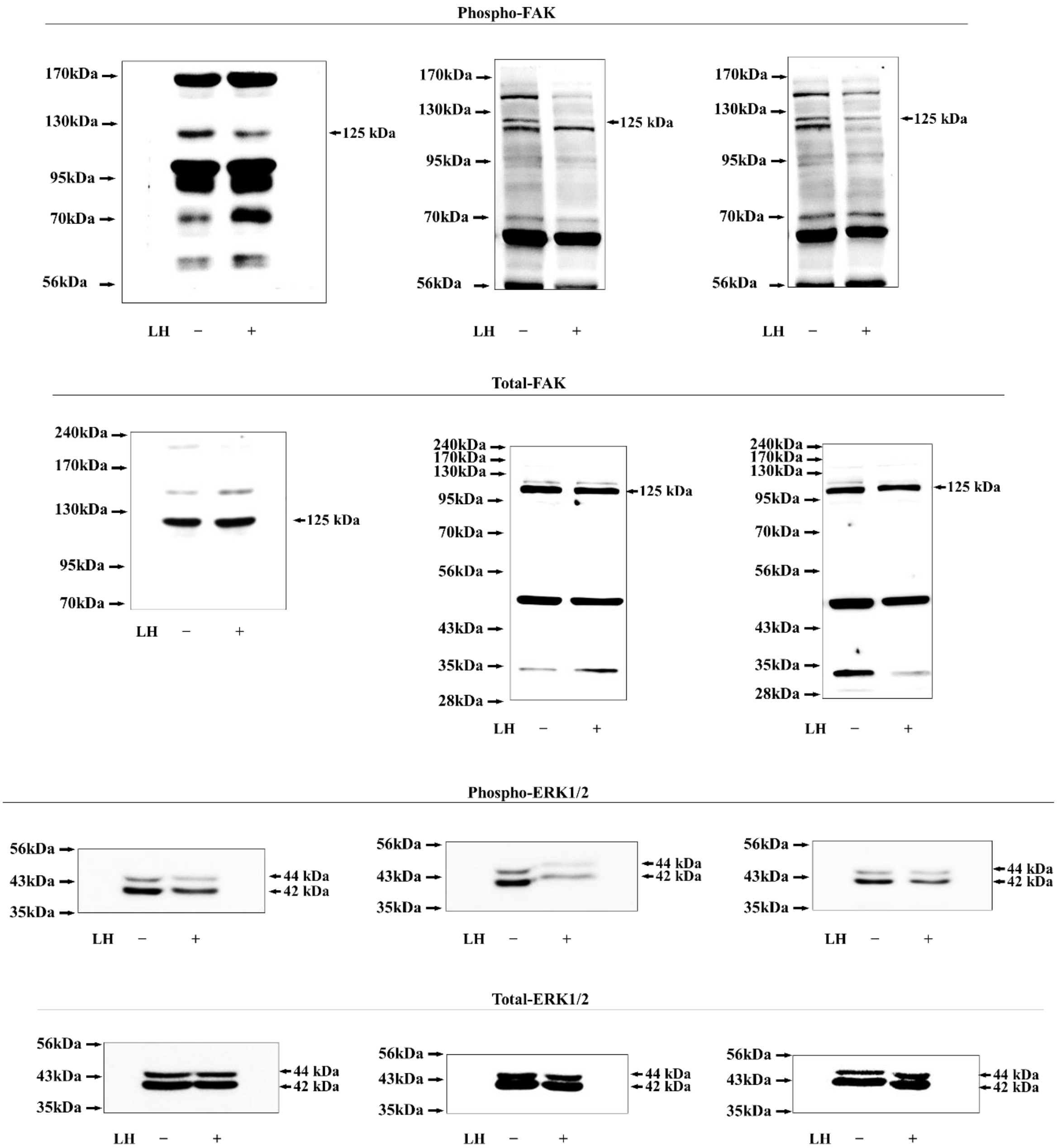

Phospho-FAK
170kDa
130kDa
95kDa
70kDa
56kDa
125 kDa
LH − +
Total-FAK
240kDa
170kDa
130kDa
95kDa
70kDa
56kDa
43kDa
35kDa
28kDa
125 kDa
LH − +
Phospho-ERK1/2
56kDa
43kDa
35kDa
44 kDa
42 kDa
LH − +
Total-ERK1/2
56kDa
43kDa
35kDa
44 kDa
42 kDa
LH − +

Figure 4D

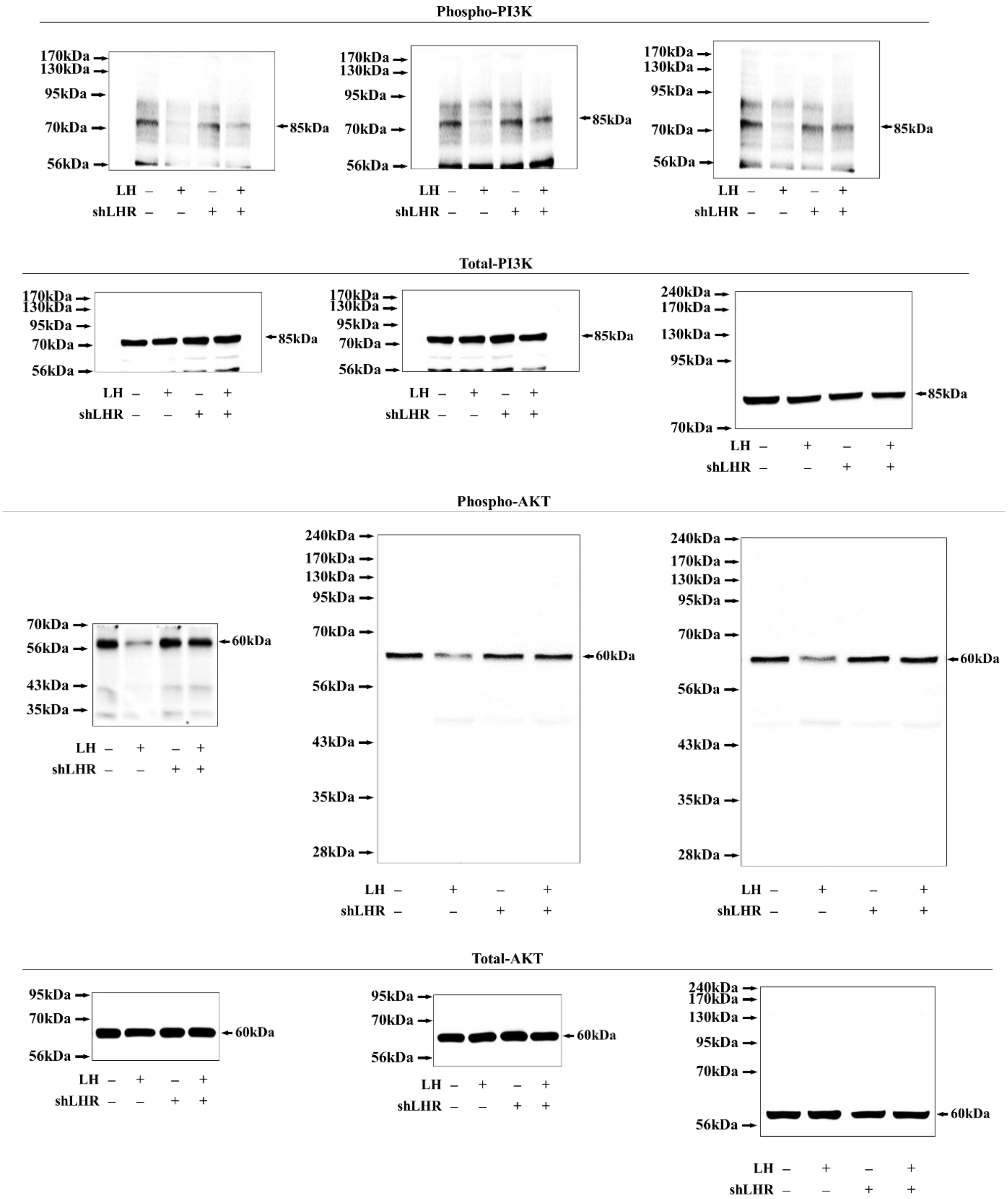
Phospho-PI3K
170kDa
130kDa
95kDa
70kDa
56kDa
85kDa
LH – + – +
shLHR – – + +
Total-PI3K
240kDa
170kDa
130kDa
95kDa
70kDa
56kDa
85kDa
LH – + – +
shLHR – – + +
Phospho-AKT
240kDa
170kDa
130kDa
95kDa
70kDa
56kDa
43kDa
35kDa
28kDa
60kDa
LH – + – +
shLHR – – + +
Total-AKT
240kDa
170kDa
130kDa
95kDa
70kDa
56kDa
60kDa
LH – + – +
shLHR – – + +

Figure 4E

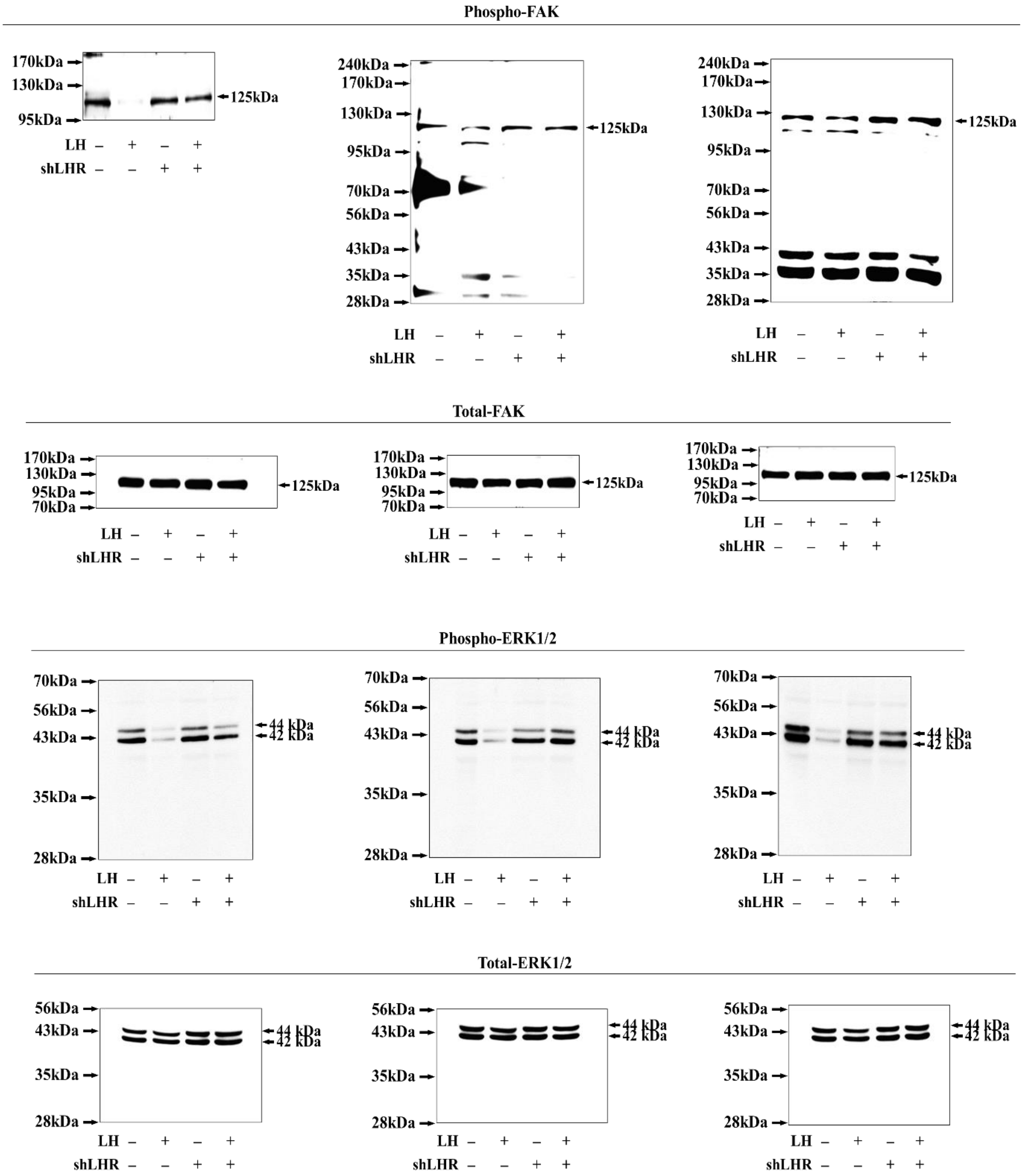
Phospho-FAK
170kDa
130kDa
95kDa
125kDa
LH – + – +
shLHR – – + +
240kDa
170kDa
130kDa
95kDa
70kDa
56kDa
43kDa
35kDa
28kDa
125kDa
Total-FAK
170kDa
130kDa
95kDa
70kDa
125kDa
Phospho-ERK1/2
70kDa
56kDa
43kDa
35kDa
28kDa
44 kDa
42 kDa
Total-ERK1/2
56kDa
43kDa
35kDa
28kDa
44 kDa
42 kDa

Figure 5D

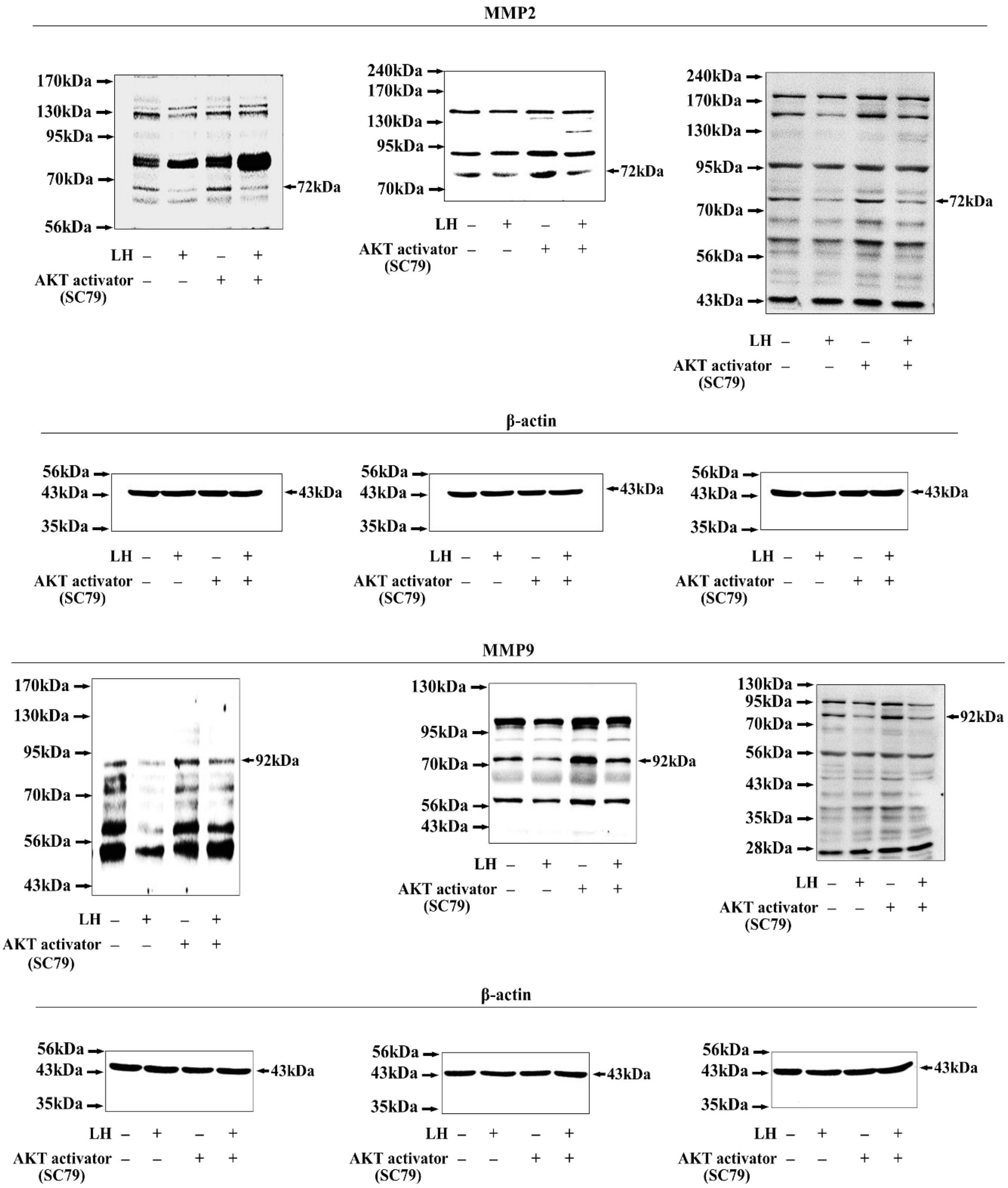
MMP2
170kDa
130kDa
95kDa
70kDa
56kDa
72kDa
LH – + – +
AKT activator – – + +
(SC79)
240kDa
170kDa
130kDa
95kDa
70kDa
72kDa
LH – + – +
AKT activator – – + +
(SC79)
240kDa
170kDa
130kDa
95kDa
70kDa
56kDa
43kDa
72kDa
LH – + – +
AKT activator – – + +
(SC79)
β-actin
56kDa
43kDa
35kDa
43kDa
LH – + – +
AKT activator – – + +
(SC79)
MMP9
170kDa
130kDa
95kDa
70kDa
56kDa
43kDa
92kDa
LH – + – +
AKT activator – – + +
(SC79)
130kDa
95kDa
70kDa
56kDa
43kDa
92kDa
LH – + – +
AKT activator – – + +
(SC79)
130kDa
95kDa
70kDa
56kDa
43kDa
35kDa
28kDa
92kDa
LH – + – +
AKT activator – – + +
(SC79)
β-actin
56kDa
43kDa
35kDa
43kDa
LH – + – +
AKT activator – – + +
(SC79)

Figure 6D

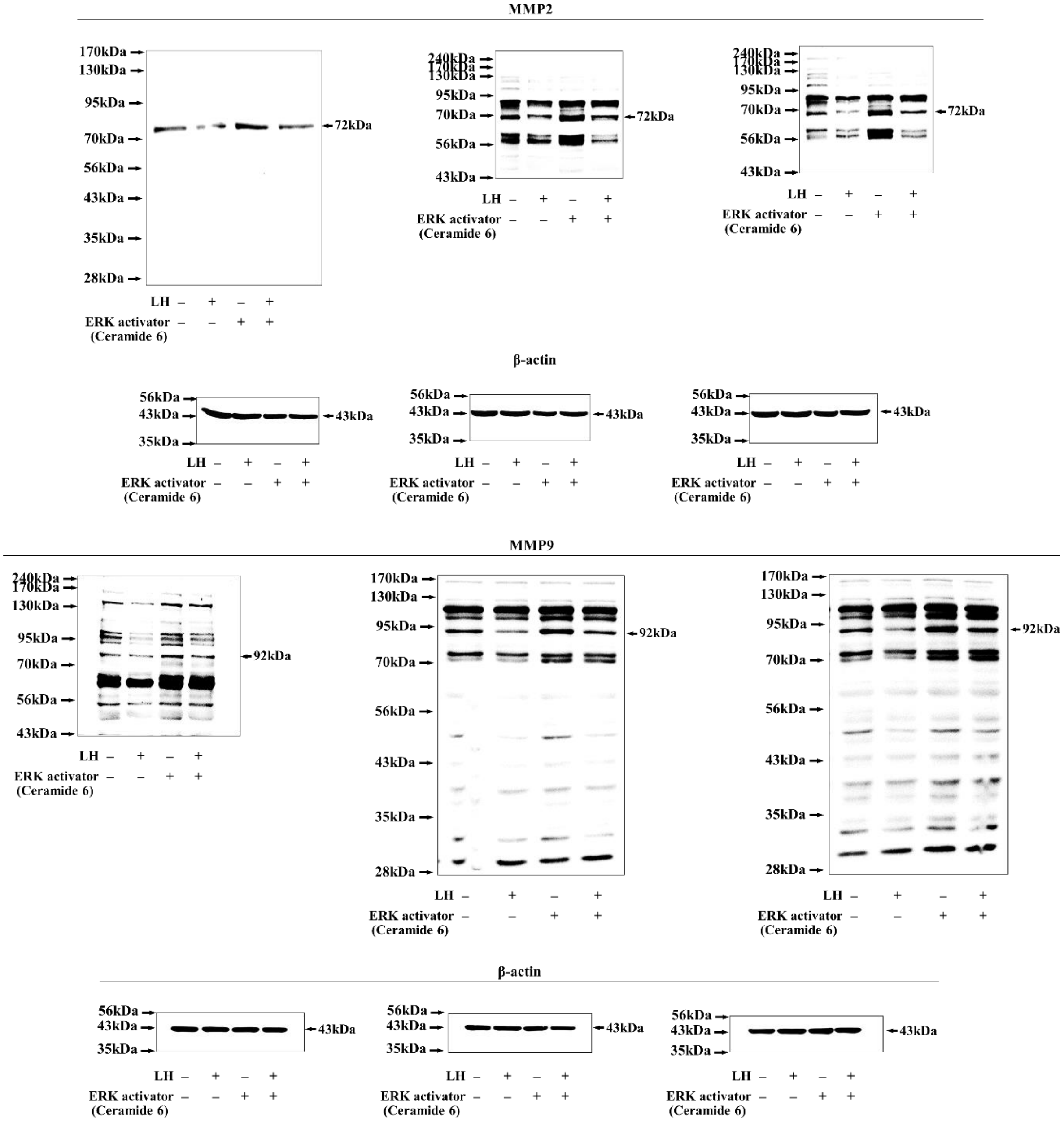
MMP2
170kDa
130kDa
95kDa
70kDa
56kDa
43kDa
35kDa
28kDa
240kDa
72kDa
LH – + – +
ERK activator – – + +
(Ceramide 6)
β-actin
43kDa
MMP9
92kDa

Figure 8F

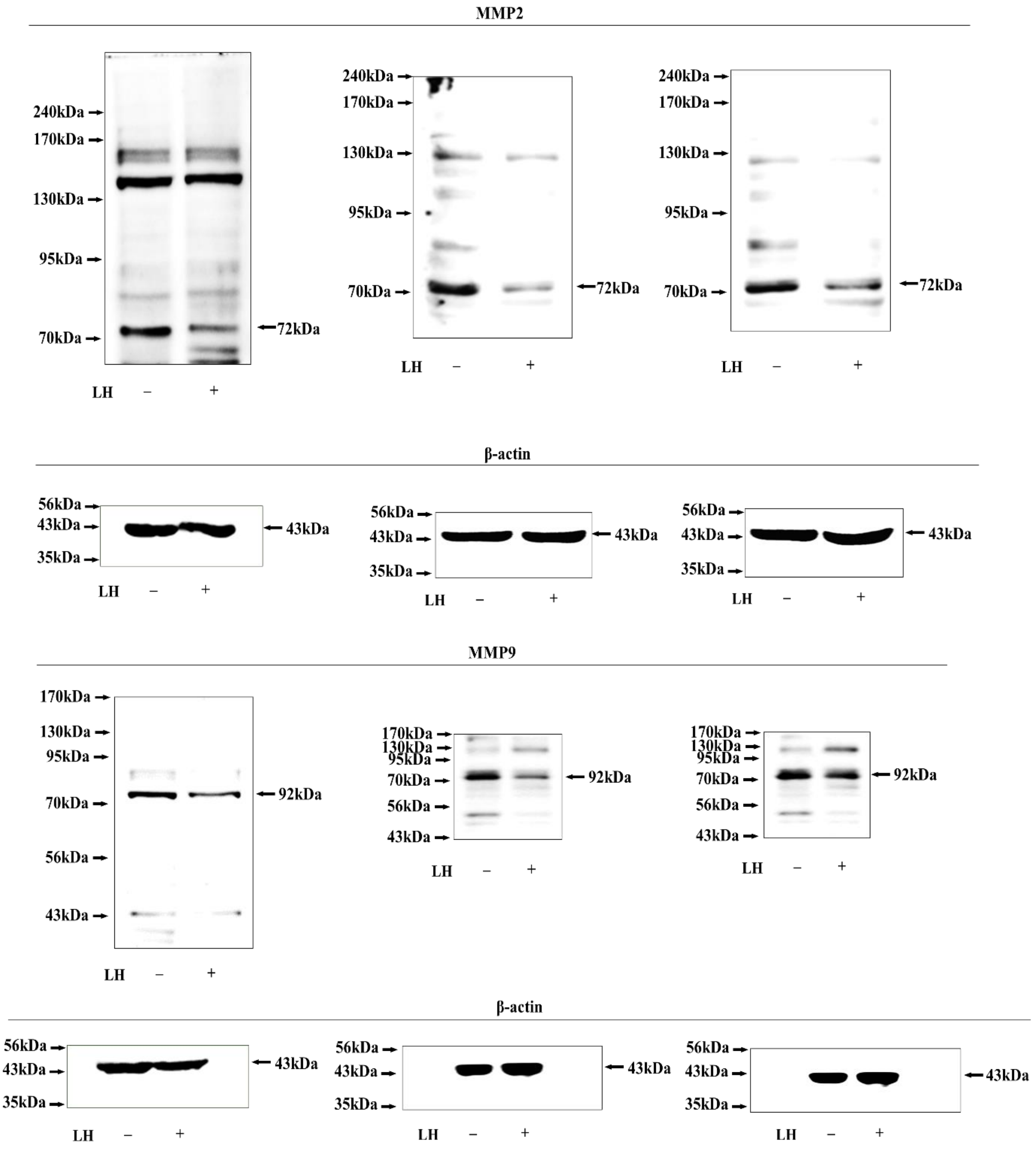
MMP2
240kDa
170kDa
130kDa
95kDa
70kDa
72kDa
LH – +
β-actin
56kDa
43kDa
35kDa
43kDa
LH – +
MMP9
170kDa
130kDa
95kDa
70kDa
56kDa
43kDa
92kDa
LH – +
β-actin
56kDa
43kDa
35kDa
43kDa
LH – +

Supplementary figure 2C

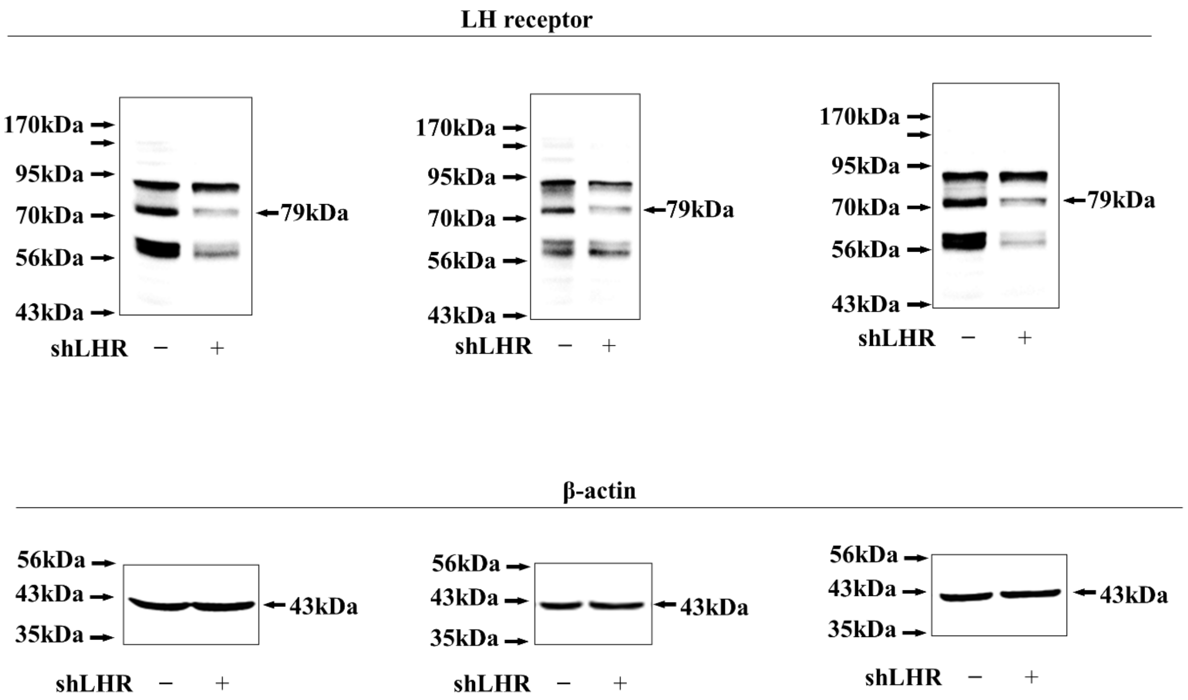

Supplementary figure 3D

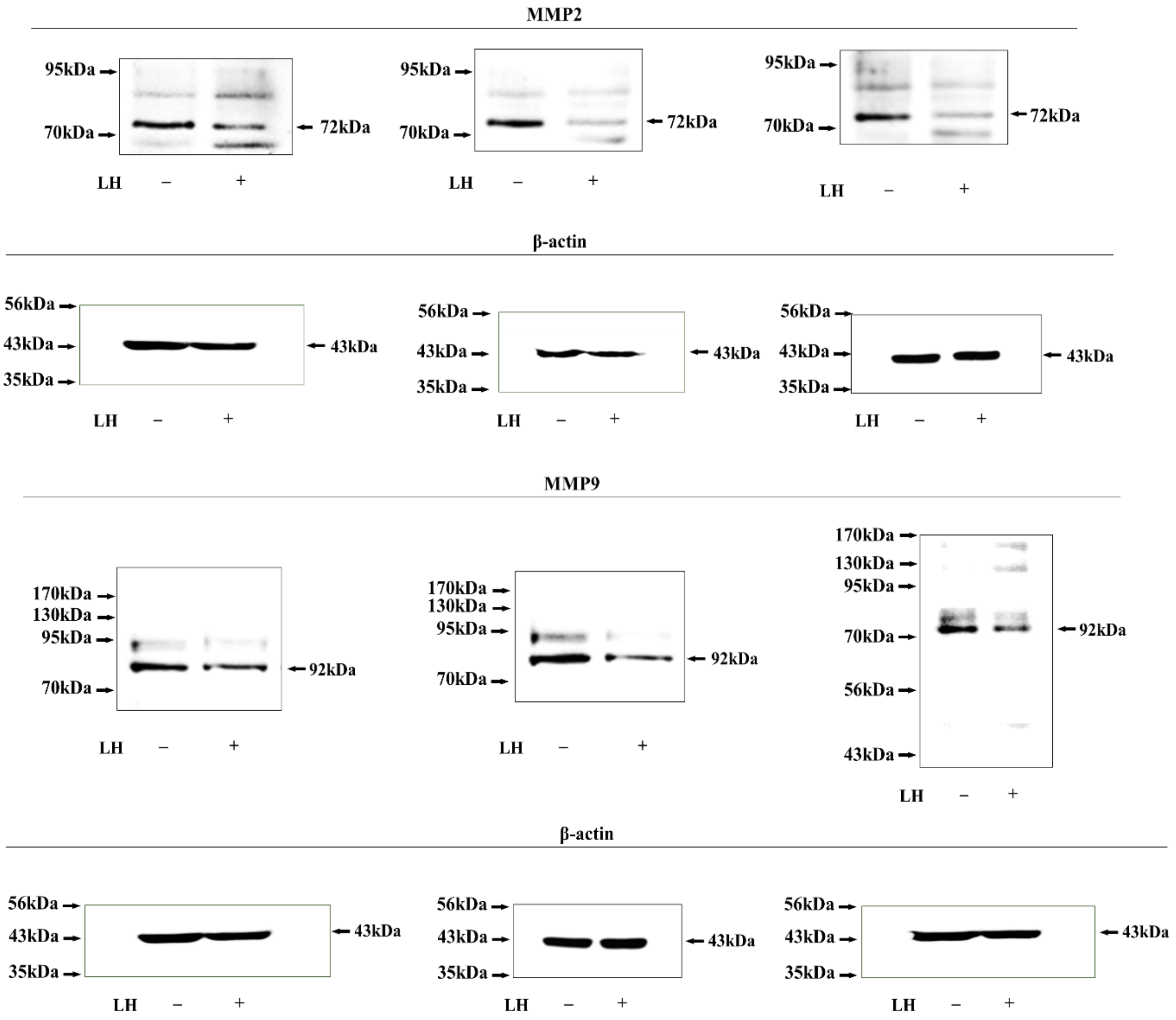

Supplementary figure 4D

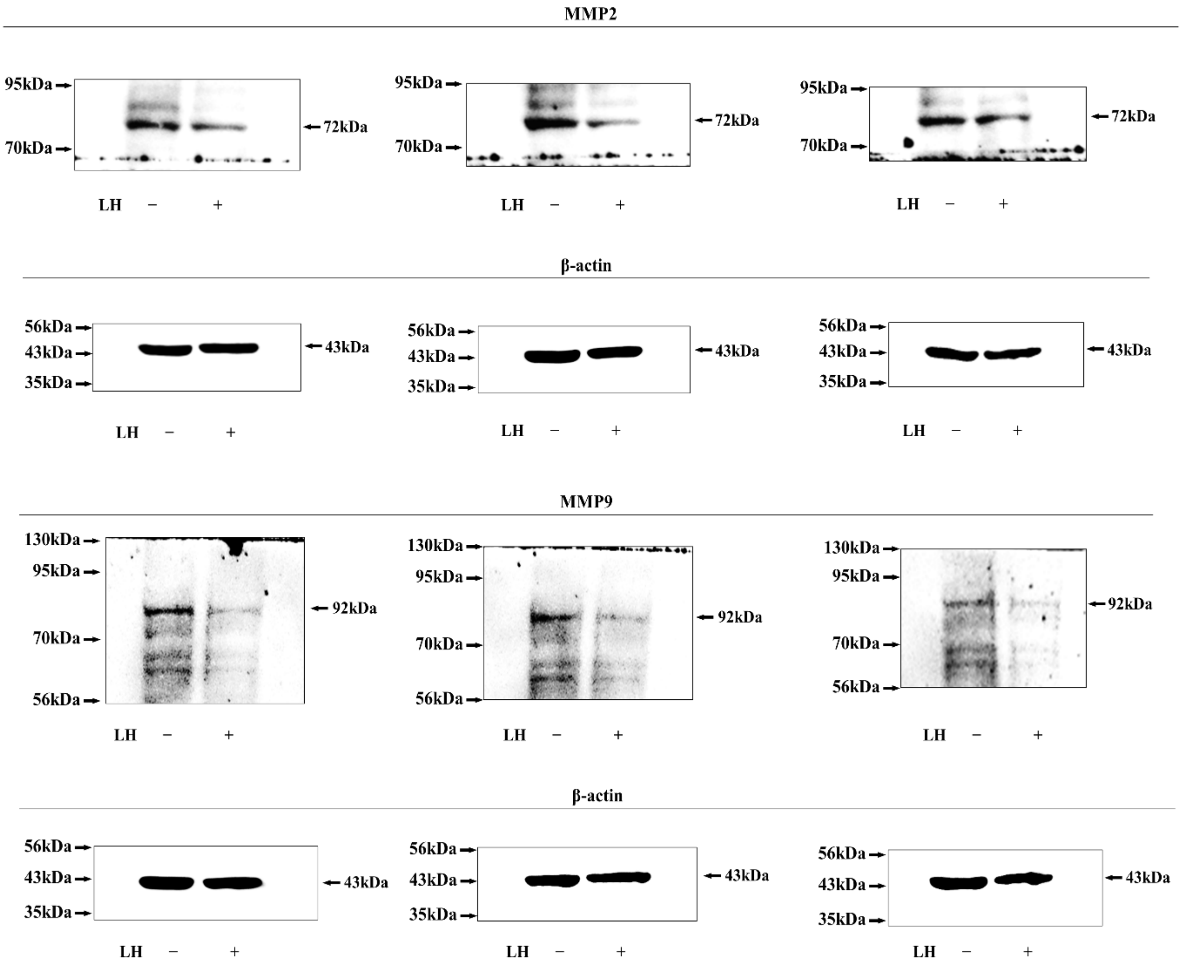

Supplement: Supplementary file 4 — Original Data File [file 41419_2022_5054_MOESM4_ESM.pdf]
